# Re-Examining the Automaticity and Directionality of the Activation of the Spatial-Valence "Good is Up" Metaphoric Association

**DOI:** 10.1371/journal.pone.0123371

**Published:** 2015-04-13

**Authors:** Yanli Huang, Chi-Shing Tse

**Affiliations:** Department of Educational Psychology, The Chinese University of Hong Kong, Hong Kong, China; The University of Nottingham, UNITED KINGDOM

## Abstract

According to the Conceptual Metaphor Theory, people understand abstract concepts depending on the activation of more concrete concepts, but not vice versa. The present research aims to investigate the role of directionality and automaticity regarding the activation of the conceptual metaphor “good is up”. Experiment 1 tested the automaticity of the spatial-to-valence metaphoric congruency effect by having participants judge the valence of a positive or negative word that appeared either at the top or at the bottom of the screen. They performed the task concurrently with a 6-digit verbal rehearsal task in the working-memory-load (WML) blocks and without this task in the non-WML blocks. The spatial-to-valence metaphoric congruency effect occurred for the positive words in the non-WML blocks (i.e., positive words are judged more quickly when they appeared at the top than at the bottom of the screen), but not in the WML blocks, suggesting that this metaphoric association might not be activated automatically. Experiments 2-6 investigated the valence-to-spatial metaphoric association and its automaticity. Participants processed a positive or negative prime, which appeared at the center of the screen, and then identified a letter (p/q) that subsequently appeared at the top or bottom of the screen. The valence-to-spatial metaphoric congruency effect did not occur in the WML (6-digit verbal rehearsal) or non-WML blocks, whether response modality to the prime was key-press or vocal, or whether the prime was a word or a picture. The effect only unexpectedly occurred when the task was simultaneously performed with a 4-dot-position visuospatial rehearsal task. Nevertheless, the data collapsed across multiple experiments showed a null valence-to-spatial metaphoric congruency effect, suggesting the absence of the valence-to-spatial metaphoric association in general. The implications of the current findings for the Conceptual Metaphor Theory and its alternatives are discussed.

## Introduction

How an abstract concept is mentally represented is one of the most important research questions in cognitive psychology. Unlike concrete concepts, abstract concepts do not have physical referents in the real world, such as valence, time, number, and morality. In daily language, we often express abstract concepts in terms of concrete concepts. For example, *I am feeling down* is used to express sad mood, time is described as an object that flows, and *thumbs up* is given when something is good. These are called *metaphors*, a figure of speech in linguistics, which is used to describe a concept by another apparently unrelated concept. The Conceptual Metaphor Theory [1,2] posits that metaphors are not only a linguistic phenomenon, but can also reflect how abstract concepts are represented in terms of more concrete, physically embodied concepts (see also [3], for a similar view). There are two possibilities: concrete concepts and abstract concepts are associated via metaphoric links (metaphoric *association*), or abstract concepts are represented in terms of concrete concepts (metaphoric *representation*) (see [4, 5] for a similar view). While it may be important to test whether the effect of conceptual metaphors could be attributed to the metaphoric association, metaphoric representation, or both, the current study was not designed to achieve that. The tasks adopted in the present study, which were directly adapted from Meier and Robinson [6], could measure the effects of metaphoric association and/or representation and our findings could be explained by the effects of metaphoric association and/or representation. Nevertheless, in this article we follow Meier and Robinson [6] and describe the effect obtained in our experiments as “an effect of metaphoric association”.

Conceptual metaphors are learned based on the conflation of conceptual domains in everyday life [1]. Concrete concepts are first acquired through sensorimotor experience. Then, the sensorimotor experience of concrete concepts can be used to represent abstract concepts via the development of metaphoric associations [7]. More specifically, when the sensorimotor experience co-occurs with the abstract concept repeatedly, two related brain areas are often activated together, such that overlapped neural mappings can be formed between the brain areas responsible for the metaphoric association between abstract and concrete concepts [8]. Therefore, the metaphoric association reflects a deeper representational, rather than merely linguistic, link between concrete and abstract concepts. For example, the vertical dimension of space is often used to express affective states (that is, positive state is described as *up* and negative state is described as *down*). This association between vertical information and valence is grounded on the repeated experience of affective state with verticality (e.g., happiness with upright posture versus sadness with a stooped posture).

In the present study, we aimed to evaluate the directionality and automaticity for the processing of the spatial-valence metaphoric association. Lakoff and Johnson [2] stated that metaphors are conceptual mappings between two domains. Source domain, often referring to the more concrete concept, provides a conceptual source with direct physical experience to target domain, which typically refers to the more abstract concept. The directionality means that the metaphoric associations are activated from source domains (concrete concepts) to target domains (abstract concepts), but not vice versa. The metaphoric congruency effect occurs when peoples’ processing of abstract concepts is faster and/or more accurate after they process a congruent concrete concept (e.g., *upward*-*good* in the spatial-to-valence metaphoric association) than after they process an incongruent concrete concept (e.g., *downward-good* in the spatial-to-valence metaphoric association). The metaphoric associations from source domains to target domains are characterized as image schemas, which structure and provide the sensorimotor grounding to abstract concepts. The automaticity means that the activation of image schemas when processing abstract concepts occurs independently of task demands and does not require any attentional resource. If the activation is automatic, the metaphoric congruency effect should be about the same magnitude whether or not people perform a secondary task that occupies their attentional resource, while processing the concrete and abstract concepts. The notions of directionality and automaticity regarding the metaphoric association have recently been investigated in the literature.

### Directionality of metaphoric association

The directionality can be divided into three levels: bidirectional, unidirectional, and asymmetric. The bidirectional view (e.g., [9]) holds that the effect of metaphoric association can occur in both concrete-to-abstract and abstract-to-concrete directions. The unidirectional view (e.g., Conceptual Metaphor Theory [1,2,8]) postulates that the effect of metaphoric association only occurs in the concrete-to-abstract direction, but not in the other way around because the sensorimotor experience of concrete concepts is useful to represent abstract concepts, but not vice versa. This view is consistent with Piaget and Inhelder’s [10] idea that sensorimotor representations of concrete concepts develop earlier and can provide scaffolding to the later processing of abstract thought (see also [11]). Santiago et al. [12] also analogized a metaphoric association as the Empire State Building. The more concrete concepts, like lower floors, provide support for the understanding of the more abstract concepts, like upper floors, but not the other way around. The asymmetric view (e.g., [13]) argues that the effect of metaphoric association can occur in both directions, with the strength in one direction (often concrete-to-abstract) being stronger than the other (often abstract-to-concrete). The critical difference between asymmetric and unidirectional views is whether the activation of the abstract concepts can have an effect on the understanding of concrete concepts. By *asymmetric*, the activation of concrete concepts has a stronger effect on the understanding of abstract concepts than vice versa. By *unidirectional*, only the activation of concrete concepts has an effect on the understanding of abstract concepts, but not vice versa. In other words, unidirectionality can be regarded as the extreme case of asymmetry.

Some researchers reported evidence that supported the asymmetric nature of some metaphoric associations. For example, in Boroditsky [14] the spatial-time metaphoric congruency effect occurred in the spatial-to-time direction—the answer to an ambiguous temporal question (e.g., “Next Wednesday’s meeting has been moved forward two days”. Please indicate to which day the meeting had been rescheduled”) depended on whether the spatial prime was ego-moving or object-moving. In contrast, such effect did not occur in the time-to-spatial direction—the answer to an ambiguous spatial question did not depend on the type of temporal prime. Boot and Pecher [15] examined the closeness-similarity metaphoric association and reported that judgments on the similarity of two color patches were affected by the distance between them, but decisions to the distance between two color patches were not affected by their similarity. However, because the effect of these metaphoric associations occurred only in one direction, but not in the other, these findings could indeed suggest that spatial-time and closeness-similarity metaphoric associations were unidirectional, rather than merely asymmetric, in nature.

Other researchers demonstrated the bidirectionality of some metaphoric associations, such as upward-powerful [16], cleanliness-morality [17], coldness-social exclusion [18], and weight-importance [19]. For instance, Giessner and Schubert [16] showed that the vertical position of a picture of a person on a screen could influence participants’ power judgments (e.g., the person appearing at the top of the screen was judged to be more powerful than the one appearing at the bottom). Information about the power of a person could also influence participants’ vertical positioning (e.g., the picture depicting a “powerful” person was judged to be higher than the one depicting a “less powerful” person).

Meier and Robinson [6] found that valence judgments were faster when positive (negative) words were presented in an upper (lower) vertical position (i.e., metaphorically congruent condition) in Study 1, indicating that participants’ valence judgments could be influenced by the primed spatial information. In Study 2, participants judged the valence of a centrally presented word before discriminating the letter (p or q) that appeared at either the top or the bottom of the screen. After judging a positive (vs. negative) word, participants were faster to discriminate the letter that appeared at the top (vs. bottom) of the screen, indicating that participants’ attention (which could directly contribute to the performance in the letter discrimination) could be biased by the primed valence information. In Study 3, Meier and Robinson reversed the sequence of events in Study 2, such that participants first determined the location (up/down) of a spatial prime (+++) and then judged the valence of a centrally presented word. However, participants’ valence judgments were not affected by their preceding spatial judgments in this study. Meier and Robinson [6] argued that Studies 2 and 3’s results demonstrated the unidirectional valence-to-spatial metaphoric association. However, given that the influence of spatial information on the valence judgment was indeed demonstrated in Study 1, Studies 1 and 2’s results could suggest the bidirectional spatial-valence metaphoric association. Moreover, the unidirectional valence-to-spatial metaphoric association in Meier and Robinson’s [6] Studies 2 and 3 was incongruent with the Conceptual Metaphor Theory [1,2,8], which postulates that the effect of metaphoric association should occur in the concrete-to-abstract direction, rather than the other way around.

To reconcile the contradictory findings between Meier and Robinson’s [6] Studies 1 and 3, one could suggest that the spatial-to-valence metaphoric congruency effect might be modulated by task demand. Specifically, spatial information influences valence judgments only when the information from two dimensions were represented as a compound cue (i.e., a positive/negative word appears at the top/bottom of the screen), but not when they were seperately presented as a prime and a target (i.e., a positive/negative word appears at the center of the screen after being primed by the “+++” sign presented at the top or bottom of the screen). However, this would leave unanswered why the effect of valence-to-spatial association could still occur when the information from two dimensions were seperately presented as a prime and a target in Meier and Robinson’s [6] Study 2. On the other hand, it was possible that the task used in their Study 3 might have a confound with an attentional bias. After determining the top (or bottom) location of the spatial prime (+++), participants needed to shift their attention downward (or upward) towards the center of the screen and judged the word valence. Thus, the effect of the spatial information of the prime on the valence judgment might have been eliminated or attenuated by the attention shifting in the opposite direction.

Given the controversy regarding the directionality of the activation of metaphoric associations, we aimed at further testing the directionality of the spatial-valence metaphoric association by using the paradigms in Meier and Robinson’s [6] Studies 1 and 2. We used their Study 1’s paradigm, instead of Study 3’s paradigm, to test the effect of spatial-to-valence metaphoric association to avoid the potential confound in attentional bias. It is also noteworthy that we tested whether the spatial-valence metaphoric congruency effect would occur in both directions of metaphoric association, rather than comparing the magnitudes of the metaphoric congruency effect that occurred in one direction vs. in the other direction. Hence, we focus on the directionality, rather than the symmetry, of metaphoric association and use the term of unidirectional vs. bidirectional, rather than asymmetric vs. symmetric, to describe the relationship between the spatial and valence concepts in the following sections.

### Automaticity of metaphoric association

According to Conceptual Metaphor Theory [1,2,8], concrete concepts are activated automatically when people process abstract concepts [20]. Meier et al. [21] investigated the automaticity for the effect of brightness-valence metaphoric associations by adopting a Stroop-like paradigm. Participants judged the valence (positive vs. negative) of affective words that were presented randomly in either black or white. Meier et al. [21] found that participants’ responses were faster and more accurate when the brightness (white and black) of the word was matched with its valence (positive and negative, respectively) than when they were mismatched. This was so even when the brightness of words did not provide any information for participants’ responses in their valence judgments, suggesting that the brightness-valence metaphoric association was activated automatically. Evidence for the automaticity was also obtained for other conceptual metaphors, such as spatial-power [22], spatial-valence [6], pitches-valence [23], and closeness-similarity [15].

In contrast, some other studies showed that the activation of metaphoric associations did not occur automatically. Santiago et al. [24] reported that the occurrence of the spatial-valence metaphoric congruency effect depended on whether participants’ attention was directed to the stimuli dimension (valence or vertical position). Similarly, de la Vega et al. [25] found that the effect of metaphoric association between physical space (left vs. right) and valence only occurred in a task with an explicit response mapping, which linked valence to response sides. Flumini and Santiago [26] showed the left-right spatial-time metaphoric association could only be activated when the task required a temporal judgment, rather than merely a lexical decision, to the time-related word (see [27] for similar findings). All these results suggest that the metaphoric congruency effect depends on whether the task requires an activation of the relevant dimension of the target stimuli (e.g., word valence when testing the spatial-valence metaphoric association) and thus does not occur automatically.

In the present study, we used a dual-task technique to test the automaticity of the activation of spatial-valence metaphoric association. The capacity view of automaticity suggests that an automatic process demands few resources from a limited-capacity attentional system (e.g., [28–31], see [32] for a review), so the automatic process should not be affected by the working memory load (WML) that occupies attentional resources. For example, Hermans et al. [33] reported that participants who performed the affective priming task and a WML (rehearsing a set of digits) simultaneously still responded faster in congruent trials than in incongruent trials, suggesting that this priming effect occurred automatically. Kroneisen et al. [34] manipulated the WML to test the automaticity of survival processing advantage in memory (i.e., better memory for words that are encoded via considering its relevance to a survival vs. other scenarios, see also [35,36]). They found that the survival processing advantage was hampered by the WML at encoding, suggesting that this mnemonic advantage might not be automatically triggered. Gao et al. [37] found that the demand of the WML (i.e., rehearsing one digit or six digits) had no influence on the magnitude of color-naming Stroop effect. They argued that the semantic activation of the color word was an automatic process that requires few attentional resources, such that it could still interfere with the naming of the ink color even when the task was simultaneously performed with a high WML (see also [38]). Hence, the extent to which a WML undermines performance in a task reflects whether the processing involved in the task is automatically triggered. In our study, if the activation of spatial-valence metaphoric association was automatic and did not require any attentional resource, then the WML would not modulate the spatial-valence metaphoric congruency effect. If the WML reduced or eliminated the metaphoric congruency effect, this would suggest that the activation of the metaphoric association may be effortful (i.e., not automatic).

### Present research

The current study investigated the directionality and automaticity of spatial-valence metaphoric association. In Experiment 1, the Stroop-like paradigm in Meier and Robinson’s [6] Study 1 was used to test the spatial-to-valence metaphoric congruency effect. To test the automaticity of this effect, participants performed half of the trials with a verbal WML (i.e., rehearsing six sequentially presented digits in the correct order), and the other half, without a WML. In Experiment 2, we adopted the paradigm in Meier and Robinson’s Study 2 to test the metaphoric congruency effect in the other direction (i.e., valence-to-spatial). We manipulated the WML again to investigate the automaticity of valence-to-spatial metaphoric association. Experiments 3–5 further tested the valence-to-spatial metaphoric congruency effect by switching the response modality from reading aloud to pressing keys to respond to the prime words, using affective pictures rather than affective words, and using a visuospatial WML (rehearsing the order of the 4 dots that were sequentially presented in different positions on the screen), instead of verbal WML. In Experiment 6, we dropped the WML manipulation and directly tested the valence-to-spatial metaphoric congruency effect by adopting the exact same paradigm as in Meier and Robinson’s Study 2. (See Table 1 for a summary of the manipulations in Experiments 1–6.)

**Table 1:** Summary of the manipulations and findings of Experiments 1–6.

Experiment	Manipulation				Metaphoric congruency effect	
	Prime Type	Response Modality to the Prime	Target Type	WML Type	With WML	Without WML
**1 (SV)**	Fixation	—	Word	Yes (6-digit)	Absent	Present
**2 (VS)**	Word	Vocal	p/q	Yes (6-digit)	Absent	Absent
**3 (VS)**	Word	Key-press	p/q	Yes (6-digit)	Absent	Absent
**4 (VS)**	Picture	Vocal	p/q	Yes (6-digit)	Absent	Absent
**5 (VS)**	Word	Vocal	p/q	Yes (4-dot)	Present	Absent
**6 (VS)**	Word	Vocal	p/q	No WML manipulation	—	Absent

*Note*: SV = Experiment that investigates the spatial-to-valence metaphoric association; VS = Experiment that investigates the valence-to-spatial metaphoric association. In the Prime Type column, Fixation = a fixation point +++ moving from the center to the top/bottom in three steps, see Fig 1A, Word = a positive or negative word, and Picture = a positive or negative picture. In the WML Type column, 6-digit = a 6-digit rehearsal task as the verbal working memory load and 4-dot = a 4-dot-position rehearsal task as the visuospatial working memory load.

## Experiment 1

In this experiment, we examined the spatial-to-valence metaphoric congruency effect in a word valence judgment task. We expected that participants would be faster and/or more accurate when the word appeared in the metaphorically congruent position (positive words at the top and negative words at the bottom) than in the metaphorically incongruent position (negative words at the top and positive words at the bottom). We also tested whether this metaphoric congruency effect could be affected by a concurrent verbal working memory load (WML). We predicted that if the spatial-to-valence metaphoric association were activated automatically, the WML would not influence the metaphoric congruency effect; otherwise, the metaphoric congruency effect would be reduced, if not eliminated, by the WML.

### Methods

#### Ethics statement

All participants signed the informed consent form prior to their participation. All experiments reported in this article were approved by the Chinese University of Hong Kong (CUHK) Survey and Behavioral Research Ethics Committee.

#### Participants

In all experiments, participants were undergraduate or graduate students recruited from the CUHK. They participated in exchange for HK$50 (about US$6.4), reported to have normal or corrected-to-normal vision. Fifty-one students (31 females; mean age = 20.35 years, *SD =* 1.73, 50 right-handers) were recruited in Experiment 1.

#### Stimuli

To make sure the stimuli were appropriate for our HK participants, we recruited 48 CUHK students, who did not participate in any of the current experiments, to perform a normed rating task on a 6-point scale on four dimensions of 696 words (including valence, arousal, familiarity, and concreteness). Based on word length (i.e., the number of letter), Log HAL frequency [39] and the ratings of the four dimensions of the words, we selected 48 positive and 48 negative words, which were significantly different in ratings of the valence dimension (4.88, *SD* = .34 vs. 2.15, *SD* = .26, *t*(94) = 43.69, *p*<.001), but not in ratings of arousal (3.76, *SD* = .48 vs. 3.66, *SD* = .40, *t*(94) = 1.15, *p* = .25), familiarity (4.74, *SD* = .36 vs. 4.63, *SD* = .35, *t*(94) = 1.58, *p* = .12), and concreteness (3.67, *SD* = .67 vs. 3.88, *SD* = .68, *t*(94) = 1.56, *p* = .12), word length (7.00, *SD* = 1.99 vs. 6.94, *SD* = 2.23, *t*(94) = .15, *p* = .89), or Log HAL frequency (9.07, *SD* = .96 vs. 8.99, *SD* = 1.06, *t*(94) = .40, *p* = .69) (see S1 Materials for the full set of stimuli). This set of stimuli was used in Huang et al.’s [40] Study 1b, in which the spatial-to-valence metaphoric congruency effect was replicated using the paradigm in Meier and Robinson’s [6] Study 1. All 48 positive and 48 negative words were presented once in the WML blocks and once in the non-WML blocks. Specifically, the 96 words were divided equally and randomly into 4 sets of 12 positive and 12 negative words, which were assigned to 4 non-WML blocks and 4 WML blocks. There were totally 192 [= 2 × (4 × 12 + 4 × 12)] experimental trials in the eight blocks. The sequence of the two types of blocks alternated (e.g., non-WML block, WML block, non-WML block, WML block…) and was counterbalanced between participants.

#### Procedure

At the beginning of each WML block, participants were given a randomly generated 6-digit sequence and instructed to keep rehearsing the six digits in the same order overtly while performing the valence judgment task. At the end of the WML block (i.e., 24 trials), participants were asked to recall the six digits in the correct order by typing them. This WML task was similar to the verbal working memory task used in previous studies (e.g., [41,42]). At the beginning of each non-WML block, participants were told that they would not need to remember or rehearse any digits in the following block of trials. Prior to the experimental trials, participants received two practice blocks of 24 trials each (one for the non-WML block and one for the WML block), in which the positive and negative words would not be used again in the experimental trials.

**Fig 1:**
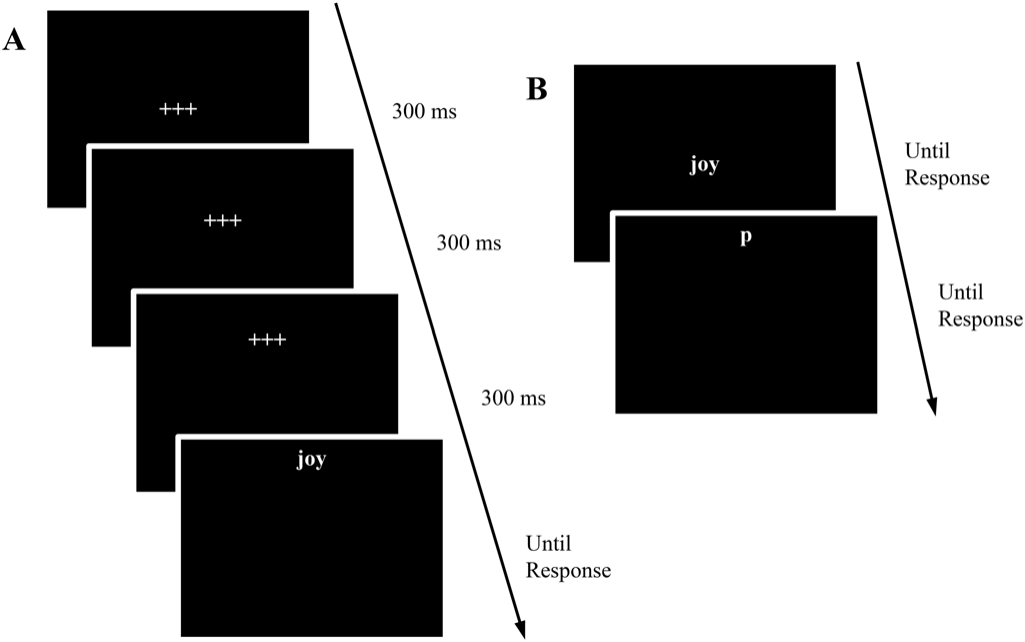
(A) A trial of the valence judgment task in Experiment 1. (B) A trial of the p/q discrimination task in Experiments 2–6.

On each trial of the valence judgment task (see Fig 1A), after the presentation of a 300-ms fixation at the center of the screen, two successive fixations appeared for 300-ms at about 1.5 inch and 3.0 inches above or below the center of the screen. Then the probe word appeared at about 4.0 inches above or below the center with the same direction as the prior two fixations. Participants needed to judge the valence (positive or negative) of the word by pressing the “A” or “L” key as quickly and as accurately as possible. A 1.5-s visual feedback message, “Incorrect”, in red appeared after the incorrect response. For correct responses, a 500-ms blank screen appeared after the participant’s response. The key assignment of the word-valence judgments was counterbalanced between participants. In each block, half of the positive and negative words were presented at the top, the other half, at the bottom of the screen, so participants could not predict word valence based on where the word appeared on the screen. In order to avoid the familiarity effect, each word appeared twice, but in different positions (top vs. bottom) on the screen in the non-WML and WML blocks, respectively.

In this paradigm, the word was presented in the same direction as the two preceding fixations. Given that the position of the two fixations and of the word may provide the spatial information that primed the processing of the word valence, one could question whether the spatial-to-valence metaphoric congruency effect, if any, could be attributed to the spatial information primed by the two fixations or by the word *per se*. However, as the goal of the current study was to test if the spatial information could prime the processing of word valence, irrespective of its source, whether the spatial information of the fixations, the spatial information of the word, or both of them triggered the spatial-to-valence metaphoric congruency effect would not affect our conclusion.

### Results

Three participants’ data (2 females) were removed due to their low accuracies in the valence judgment task (<80%), low accuracies in the WML task (<80%), or their extreme overall reaction times (RTs) (3 *SDs* from the mean). Thus, data from 48 participants were included in the final analyses. [With *N* of 48 in the current study and Cohen’s *d* of .42, which was computed based on the *F* value (6.11, i.e., *t* of 2.47) and sample size (34) reported in the Meier and Robinson’s [6] Study 1, the power to detect the effect in our Experiment 1 was .65. We used the procedure recommended by Lakens [43] to compute the Cohen’s *d*s. As we predicted a specific direction for the metaphoric congruency effect (i.e., incongruent > congruent in RT), we reported the statistical power based on *α* = .05 (one-tailed) [44].] The mean accuracy for these participants’ digit recall in the WML blocks was .99 (*SD* = .03). In RT analyses, we followed the same trimming procedure as in Meier and Robinson’s [6] Study 1. That is, we first excluded incorrect trials (4.31% of all responses), and then log-transformed RTs of the remaining trials to normalize the distribution. Trials above 2.5 *SD*s from the mean of overall RTs were replaced with 2.5-*SD* values. In accuracy analyses all accuracy data were included. (See Tables 2 and 3 for the descriptive statistics of all six experiments.)

**Table 2:** Overall cell means (in ms) (and standard deviations) in Experiments 1–6.

Block	Position	Valence	Experiment 1	Experiment 2	Experiment 3	Experiment 4	Experiment 5	Experiment 6
**Non-WML**	**Top**	**Positive**	830.85 (147.53)	597.88 (70.20)	565.02 (84.06)	590.02 (70.47)	578.78 (73.02)	592.39 (88.46)
	**Top**	**Negative**	868.94 (150.02)	614.29 (73.69)	571.46 (87.58)	613.93 (68.50)	588.65 (71.78)	608.01 (87.51)
	**Bottom**	**Positive**	873.64 (162.27)	604.82 (65.47)	577.99 (85.18)	600.94 (69.94)	578.68 (69.37)	599.64 (86.94)
	**Bottom**	**Negative**	872.86 (153.86)	619.07 (71.82)	583.65 (92.42)	617.97 (70.25)	598.67 (65.33)	618.54 (85.60)
**WML**	**Top**	**Positive**	860.63 (158.44)	591.77 (67.46)	571.32 (95.72)	592.91 (77.46)	578.15 (68.66)	—
	**Top**	**Negative**	859.48 (148.44)	612.78 (68.58)	579.30 (96.53)	615.51 (67.28)	597.84 (74.18)	—
	**Bottom**	**Positive**	855.41 (178.57)	599.29 (75.04)	592.03 (99.93)	606.36 (70.60)	593.01 (69.84)	—
	**Bottom**	**Negative**	870.56 (163.08)	624.48 (66.18)	589.92 (94.19)	622.42 (73.35)	596.95 (62.22)	—

*Note*: The cell means in each condition are computed by participants. The cell means were based on the raw data after trimming out the incorrect trials and replacing trials that were 2.5 *SD*s below or above the overall RT mean with the 2.5-*SD* values, not the log-transformed data, for the ease of interpretation.

**Table 3:** Overall cell means of accuracies (in proportion) (and standard deviations) in Experiments 1–6.

Block	Position	Valence	Experiment 1	Experiment 2	Experiment 3	Experiment 4	Experiment 5	Experiment 6
**Non-WML**	**Top**	**Positive**	0.974 (0.036)	0.975 (0.034)	0.970 (0.044)	0.979 (0.031)	0.980 (0.034)	0.975 (0.031)
	**Top**	**Negative**	0.949 (0.049)	0.960 (0.054)	0.974 (0.034)	0.964 (0.056)	0.968 (0.044)	0.964 (0.041)
	**Bottom**	**Positive**	0.959 (0.045)	0.972 (0.033)	0.968 (0.035)	0.964 (0.045)	0.973 (0.031)	0.964 (0.037)
	**Bottom**	**Negative**	0.944 (0.050)	0.957 (0.051)	0.958 (0.045)	0.971 (0.041)	0.953 (0.049)	0.956 (0.041)
**WML**	**Top**	**Positive**	0.952 (0.039)	0.971 (0.033)	0.970 (0.037)	0.967 (0.052)	0.972 (0.032)	—
	**Top**	**Negative**	0.944 (0.046)	0.963 (0.046)	0.964 (0.043)	0.965 (0.043)	0.962 (0.040)	—
	**Bottom**	**Positive**	0.976 (0.031)	0.963 (0.048)	0.978 (0.031)	0.958 (0.059)	0.969 (0.037)	—
	**Bottom**	**Negative**	0.957 (0.047)	0.962 (0.045)	0.964 (0.046)	0.962 (0.054)	0.957 (0.044)	—

*Note*: The cell means in each condition are computed by participants based on all accuracy data.

**Fig 2:**
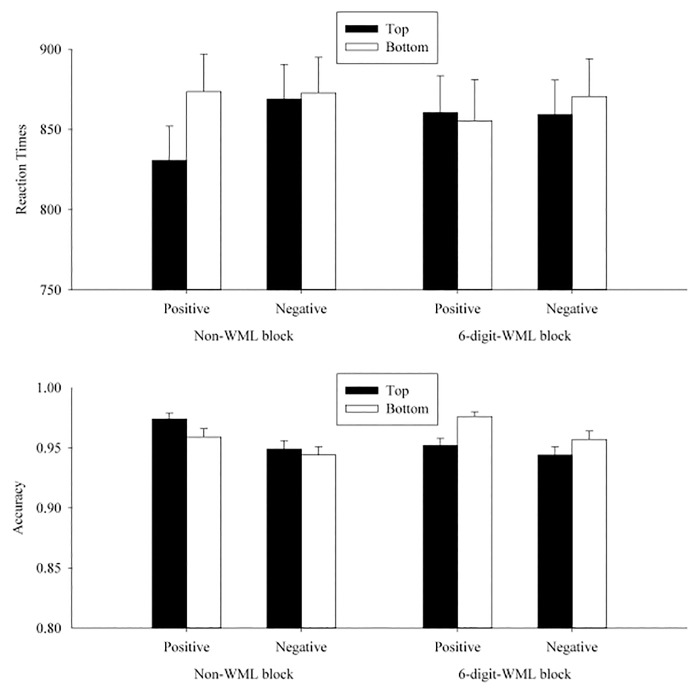
Mean RTs (upper panel) and accuracies (lower panel) in each condition on the valence judgment in Experiment 1. The error bars indicated the standard errors of the means. *Note*: The cell means illustrated in all figures were averaged based on the RTs, instead of log-transformed RTs, with the trimming procedure described in the main text.

A 2 (WML: WML vs. non-WML) × 2 (position: top vs. bottom) × 2 (valence of the target word: positive vs. negative) ANOVA was conducted for participants’ transformed RT and accuracy, separately. The significance level was set at .05, two tailed, for all the tests. We report both analyses with participants (*F*_1_) and with items (*F*_2_) as the random factor. The effect sizes of *F* statistics are reported in partial eta-squared (*η*_*p*_^*2*^). For the sake of simplicity, in the following we report the critical three-way (WML × position × valence) interaction and the follow-up simple effect analyses. This data analytic procedure was used in all experiments. (See Table 4 for the omnibus ANOVA results for the transformed RTs and accuracies in participants and item analyses and Fig 2 for the graphic illustration of the cell means in Experiment 1.)

**Table 4:** Omnibus Analyses of Variance for log-transformed mean response times (RTs, in ms) and accuracies (in proportion) in Experiment 1.

	By participant								By item							
	RT				Accuracy				RT				Accuracy			
	*MS*_*E*_	*F*_*1*_	*p*	*η*_*p*_^*2*^	*MS*_*E*_	*F*_*1*_	*p*	*η*_*p*_^*2*^	*MS*_*E*_	*F*_*2*_	*p*	*η*_*p*_^*2*^	*MS*_*E*_	*F*_*2*_	*p*	*η*_*p*_^*2*^
**WML**	.001	.01	.91	<.001	.001	.03	.86	.001	.001	.01	.92	<.001	.002	.01	.93	<.001
**Position**	.001	5.31	.03	.10	.001	1.31	.26	.03	.001	1.19	.28	.006	.002	.35	.55	.002
**Valence**	.001	3.29	.08	.07	.003	10.57	.002	.18	.001	1.28	.26	.007	.002	5.76	.02	.03
**WML × Position**	<.001	6.37	.02	.12	.002	11.63	.001	.20	.001	1.55	.22	.008	.002	3.86	.05	.02
**WML × Valence**	.001	.90	.35	.02	.001	.98	.33	.02	.001	.19	.67	.001	.002	.22	.64	.001
**Position × Valence**	.001	1.28	.26	.03	.001	.004	.95	<.001	.001	.29	.59	.002	.002	.001	.98	<.001
**WML × Position × Valence**	.001	7.86	.01	.14	.002	1.46	.23	.03	.001	1.52	.22	.008	.002	.61	.44	.003

*Note*: The degree of freedom in the participant analyses was (1, 47). The degree of freedom in the item analyses was (1, 184).

In the RT analyses, the WML × position × valence interaction was significant by participants, *F*_1_(1, 47) = 7.86, *MS*_*E*_ = .001, *p* = .01, *η*_*p*_^2^ = .14; *F*_2_(1, 184) = 1.52, *MS*_*E*_ = .001, *p* = .22, *η*_*p*_^2^ = .008. Follow-up analyses showed that in the non-WML blocks, the valence × position interaction was significant by participants, *F*_1_(1,47) = 7.16, *MS*_*E*_ = .001, *p* = .01, *η*_*p*_^*2*^ = .13; *F*_2_(1,92) = 1.63, *MS*_*E*_ = .001, *p* = .21, *η*_*p*_^*2*^ = .02. Simple effect analyses showed that positive words were responded to faster when they appeared at the top of the screen than at the bottom, *F*_1_(1,47) = 13.59, *p* = .001, *η*_*p*_^*2*^ = .22; *F*_2_(1,92) = 4.38, *p* = .04, *η*_*p*_^*2*^ = .05, demonstrating a metaphoric congruency effect. However, there was no significant effect of position for negative words, *F*_1_(1,47) = .18, *p* = .67, *η*_*p*_^*2*^ = .004; *F*_2_(1,92) = .08, *p* = .77, *η*_*p*_^*2*^ = .001. On the other hand, in the WML blocks the valence × position interaction was not significant, *F*_1_(1,47) = 1.73, *MS*_*E*_<.001, *p* = .19, *η*_*p*_^*2*^ = .04; *F*_2_(1,92) = .23, *MS*_*E*_ = .001, *p* = .63, *η*_*p*_^*2*^ = .003, suggesting that the metaphoric congruency effect in RT was eliminated when participants performed the WML and valence judgment tasks simultaneously. In accuracy analyses, neither the three-way interaction, *F*_1_(1,47) = 1.46, *MS*_*E*_ = .002, *p* = .23, *η*_*p*_^*2*^ = .03; *F*_2_(1,184) = .61, *MS*_*E*_ = .002, *p* = .44, *η*_*p*_^*2*^ = .003, nor the position × valence interaction was significant, *F*_1_(1,47) = .004, *MS*_*E*_ = .001, *p* = .95, *η*_*p*_^*2*^<.001; *F*_2_(1,184) = .001, *MS*_*E*_ = .002, *p* = .98, *η*_*p*_^*2*^<.001.

Despite the high accuracy of participants’ digit recall in the WML blocks (99%), we conducted a 2 (position) × 2 (valence) ANOVA only on the data from the WML blocks in which digit recall was correct. In RT analyses, the main effect of position was not significant, *F*_*1*_(1,47) = .003, *MS*_*E*_<.001, *p* = .96, *η*_*p*_^*2*^<.001; *F*_*2*_(1,92) = .01, *MS*_*E*_ = .001, *p* = .91, *η*_*p*_^*2*^<.001. The main effect of valence was not significant, *F*_*1*_(1,47) = 1.26, *MS*_*E*_ = .001, *p* = .27, *η*_*p*_^*2*^ = .03; *F*_*2*_(1,92) = .27, *MS*_*E*_ = .001, *p* = .61, *η*_*p*_^*2*^ = .003. The position × valence interaction was not significant, *F*_*1*_(1,47) = 1.11, *MS*_*E*_<.001, *p* = .30, *η*_*p*_^*2*^ = .02; *F*_*2*_(1,92) = .10, *MS*_*E*_ = .001, *p* = .76, *η*_*p*_^*2*^ = .001. In accuracy analyses, the main effect of position was significant by participants, *F*_*1*_(1,47) = 7.10, *MS*_*E*_ = .002, *p* = .01, *η*_*p*_^*2*^ = .13; *F*_*2*_(1,92) = 2.52, *MS*_*E*_ = .002, *p* = .12, *η*_*p*_^*2*^ = .03. The main effect of valence was not significant, *F*_*1*_(1,47) = 3.23, *MS*_*E*_ = .002, *p* = .08, *η*_*p*_^*2*^ = .06; *F*_*2*_(1,92) = 1.27, *MS*_*E*_ = .002, *p* = .26, *η*_*p*_^*2*^ = .01. The position × valence interaction was not significant, *F*_*1*_(1,47) = 2.03, *MS*_*E*_ = .001, *p* = .16, *η*_*p*_^*2*^ = .04; *F*_*2*_(1,92) = .55, *MS*_*E*_ = .002, *p* = .46, *η*_*p*_^*2*^ = .01.

### Discussion

Experiment 1 successfully replicated the spatial-to-valence metaphoric congruency effect in the non-WML blocks in the participant analyses, consistent with the results of Meier and Robinson’s [6] Study 1. Participants responded faster when positive words appeared at the top than at the bottom. This spatial-to-valence metaphoric congruency effect disappeared in the WML blocks, suggesting that the spatial-to-valence metaphoric association might not be activated automatically in the valence judgment task.

It is noteworthy that the three-way (WML × position × valence) interaction and the two-way (valence × position) interaction in the non-WML blocks did not approach statistical significance in the item analyses (*p* = .22 and *p* = .21, respectively). Hence, the finding that the metaphoric congruency effect was modulated by WML might not necessarily generalize across our current stimuli. In fact, Dudschig et al. [45] recently reported that the effect of metaphoric association between vertical position and valence occurred only for specific valence words that are related to bodily postures. This suggests that instead of a general mapping between space and *all* valence words, the effect of this metaphoric association may be modulated by some characteristics of the valence words. Future studies should further identify these specific characteristics in order to clarify the mapping in the spatial-valence metaphoric association.

In addition, one could argue that the absence of the valence-to-spatial metaphoric congruency effect in the WML blocks might be attributed to the possibility that verbal processing during valence judgments was disrupted by verbal WML. Due to the interference of verbal WML, it is possible that participants might not have been able to process the word valence. If this were so, one would predict the main effect of WML on participants’ RT and/or accuracy. That is, participants would show generally worse performance on valence judgments in the WML blocks than in the non-WML blocks, if verbal WML disrupted their processing of word valence. However, we did not obtain the main effect of WML in RT, *F*_*1*_(1,47) = .01, *MS*_*E*_ = .001, *p* = .91, *η*_*p*_^*2*^<.001 and *F*_*2*_(1,184) = .01, *MS*_*E*_ = .001, *p* = .92, *η*_*p*_^*2*^<.001, or in accuracy, *F*_*1*_(1,47) = .03, *MS*_*E*_ = .001, *p* = .86, *η*_*p*_^*2*^ = .001 and *F*_*2*_(1,184) = .01, *MS*_*E*_ = .002, *p* = .93, *η*_*p*_^*2*^<.001. Rather, the WML × valence × position interaction suggested that verbal WML disrupted the activation of the spatial-to-valence metaphoric association, rather than the activation of word valence *per se*, in the valence judgment task.

## Experiment 2

In this experiment, we investigated the valence-to-spatial metaphoric congruency effect. Following the paradigm in Meier and Robinson’s [6] Study 2 paradigm, in each trial, a positive or negative prime word first appeared at the center of the screen and participants needed to judge the valence of the word by vocal response (i.e., saying “positive” or “negative”). Then, a letter “p” or “q” appeared at either the top or bottom of the screen, and participants needed to judge its identity (p or q) by pressing the corresponding key on the keyboard. If there was a valence-to-spatial metaphoric association, when participants judged the valence of a word, the spatial information (up or down) would be activated, leading to participants’ upward or downward attentional bias. As a result, their subsequent p/q discrimination would be faster when the letter appeared in the metaphorically congruent position (e.g., at the top following the valence judgment of a positive word) than in the metaphorically incongruent position (e.g., at the bottom following the valence judgment of a positive word). As we obtained the spatial-to-valence metaphoric congruency effect in Experiment 1’s non-WML blocks, if we also found the valence-to-spatial metaphoric congruency effect in the non-WML blocks in this experiment, we could conclude that the spatial-valence metaphoric association should be bidirectional. Similar to Experiment 1, we also included WML blocks, in which participants needed to rehearse a 6-digit sequence while performing the p/q discrimination task. This could test whether a verbal WML would modulate the activation of the valence-to-spatial metaphoric association.

### Methods

#### Participants

Sixty-four students (37 females; mean age = 20.57 years, *SD =* 1.82, 63 right-handers) were recruited.

#### Stimuli and procedure

The positive and negative prime words were the same as those used in Experiment 1. Each of the 48 positive and 48 negative prime words was presented twice, once in the WML block and once in the non-WML block, with block order being counterbalanced between participants. Thus, there were 192 experimental trials in total. The WML block and non-WML block manipulations and the practice-block arrangement were identical to those in Experiment 1.

In each trial of the p/q discrimination task (see Fig 1B), a positive or negative prime word first appeared at the center of the screen. Participants were asked to judge its valence by vocally responding “positive” or “negative”. Immediately after participants’ response, a letter “p” or “q” was presented at the top or bottom of the screen. Participants needed to identify the letter by pressing the corresponding “p” or “q” key on the keyboard as quickly and as accurately as possible. A 1.5-s visual feedback message, “Incorrect”, in red was given for the incorrect response in the p/q discrimination task. For a correct response, a 500-ms blank screen appeared after participants’ responses. Following half of the positive and negative words, the letter p/q was presented at the top, and the other half, at the bottom, such that participants were not able to predict the position of p/q based on the valence of the prime word. This assignment was counterbalanced between participants.

### Results

Sixteen participants’ data (10 females) were removed due to their low accuracies in the WML task (<80%) or their extreme overall RTs (±3 *SDs* from the mean). Thus, data from 48 participants were included in the final analyses. [With *N* of 48 in the current study and Cohen’s *d* of .54, which was computed based on the *F* value (8.07, i.e., *t* of 2.84) and sample size (28) reported in the Meier and Robinson’s [6] Study 2, the power based on *α* = .05 (one-tailed) to detect the effect in our Experiment 2 was .84.] The mean accuracy for these participants’ digit recall in the WML blocks was .95 (*SD* = .05). In RT analyses, we followed the same trimming procedure as in Meier and Robinson’s [6] Study 2. That is, we first excluded incorrect trials (3.46% of all responses), and the trials involving vocal valence judgment with RT of 3 *SD*s beyond the overall mean (1.86% of all responses). Then, RTs of the remaining trials were log-transformed to normalize the distribution. Trials above 2.5 *SD*s from the mean of overall RTs were replaced with 2.5-*SD* values. In accuracy analyses all accuracy data were included.

**Fig 3:**
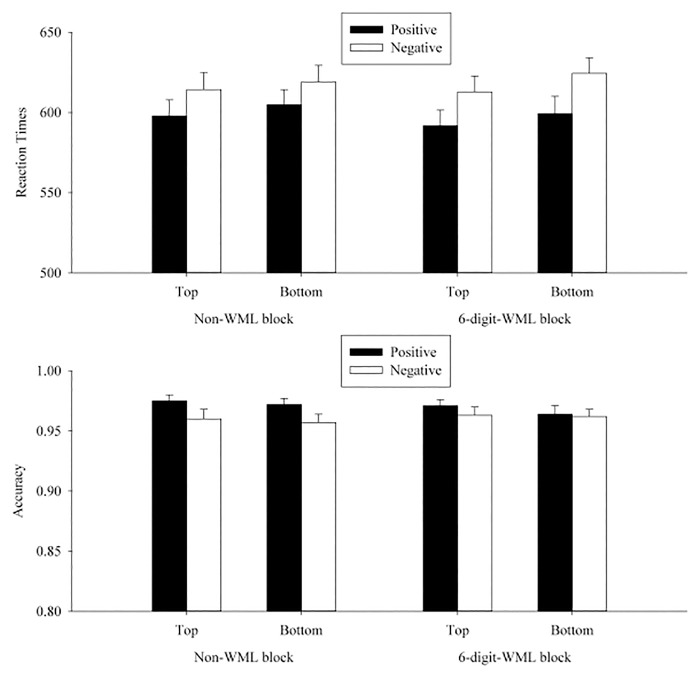
Mean RTs (upper panel) and accuracies (lower panel) in each condition on p/q discrimination in Experiment 2. The error bars indicated the standard errors of the means.

A 2 (WML: WML vs. non-WML) × 2 (position: top vs. bottom) × 2 (valence of the prime word: positive vs. negative) ANOVA was conducted for participants’ transformed RT and accuracy, separately (see Table 5 and Fig 3).

**Table 5:** Omnibus Analyses of Variance for log-transformed mean response times (RTs, in ms) and accuracies (in proportion) in Experiment 2.

	By participant								By item							
	RT				Accuracy				RT				Accuracy			
	*MS*_*E*_	*F*_*1*_	*p*	*η*_*p*_^*2*^	*MS*_*E*_	*F*_*1*_	*p*	*η*_*p*_^*2*^	*MS*_*E*_	*F*_*2*_	*p*	*η*_*p*_^*2*^	*MS*_*E*_	*F*_*2*_	*p*	*η*_*p*_^*2*^
**WML**	<.001	.65	.42	.01	.001	.10	.75	.002	<.001	1.16	.29	.01	.001	.08	.77	.001
**Position**	<.001	6.93	.01	.13	.002	.86	.36	.02	<.001	6.51	.01	.07	.001	1.03	.31	.01
**Valence**	<.001	51.03	<.001	.52	.002	5.45	.02	.10	<.001	39.13	<.001	.29	.002	5.57	.02	.06
**WML × Position**	<.001	.39	.54	.01	.001	.06	.81	.001	<.001	.64	.43	.01	.001	.03	.86	<.001
**WML × Valence**	<.001	3.23	.08	.06	.002	1.49	.23	.03	<.001	1.37	.25	.01	.001	1.77	.19	.02
**Position × Valence**	<.001	.04	.85	.001	.001	.15	.70	.003	<.001	.001	.98	<.001	.001	.19	.67	.002
**WML × Position × Valence**	<.001	.72	.40	.02	.001	.31	.58	.01	.001	.15	.70	.002	.001	.27	.60	.003

*Note*: The degree of freedom in the participant analyses was (1, 47). The degree of freedom in the item analyses was (1, 94).

In the RT analyses, neither the WML × position × valence interaction, *F*_1_(1,47) = .72, *MS*_*E*_<.001, *p* = .40, *η*_*p*_^*2*^ = .02; *F*_2_(1,94) = .15, *MS*_*E*_ = .001, *p* = .70, *η*_*p*_^*2*^ = .002, nor the position × valence interaction was significant, *F*_1_(1,47) = .04, *MS*_*E*_<.001, *p* = .85, *η*_*p*_^*2*^ = .001; *F*_2_(1,94) = .001, *MS*_*E*_<.001, *p* = .98, *η*_*p*_^*2*^<.001. In the accuracy analyses, neither the WML × position × valence interaction, *F*_1_(1,47) = .31, *MS*_*E*_ = .001, *p* = .58, *η*_*p*_^*2*^ = .01; *F*_2_(1,94) = .27, *MS*_*E*_ = .001, *p* = .60, *η*_*p*_^*2*^ = .003, nor the position × valence interaction was significant, *F*_1_(1,47) = .15, *MS*_*E*_ = .001, *p* = .70, *η*_*p*_^*2*^ = .003; *F*_2_(1,94) = .19, *MS*_*E*_ = .001, *p* = .67, *η*_*p*_^*2*^ = .002.

Despite the high accuracy of participants’ digit recall in the WML blocks (95%), we conducted a 2 (position) × 2 (valence) ANOVA only on the data from the WML blocks in which the digit recall was correct. In RT analyses, the main effect of position was significant, *F*_*1*_(1,47) = 5.74, *MS*_*E*_<.001, *p* = .02, *η*_*p*_^*2*^ = .11; *F*_*2*_(1,94) = 9.46, *MS*_*E*_<.001, *p* = .003, *η*_*p*_^*2*^ = .09. The main effect of valence was significant, *F*_*1*_(1,47) = 44.71, *MS*_*E*_<.001, *p*<.001, *η*_*p*_^*2*^ = .49; *F*_*2*_(1,94) = 27.94, *MS*_*E*_<.001, *p*<.001, *η*_*p*_^*2*^ = .23. The position × valence interaction was not significant, *F*_*1*_(1,47) = .26, *MS*_*E*_<.001, *p* = .61, *η*_*p*_^*2*^ = .006; *F*_*2*_(1,94) = .03, *MS*_*E*_<.001, *p* = .87, *η*_*p*_^*2*^<.001. In accuracy analyses, the main effect of position was not significant, *F*_*1*_(1,47) = .77, *MS*_*E*_ = .002, *p* = .39, *η*_*p*_^*2*^ = .02; *F*_*2*_(1,94) = 1.10, *MS*_*E*_ = .002, *p* = .30, *η*_*p*_^*2*^ = .01. The main effect of valence was not significant, *F*_*1*_(1,47) = .62, *MS*_*E*_ = .001, *p* = .43, *η*_*p*_^*2*^ = .01; *F*_*2*_(1,94) = .39, *MS*_*E*_ = .002, *p* = .53, *η*_*p*_^*2*^ = .004. The position × valence interaction was not significant, *F*_*1*_(1,47) = .57, *MS*_*E*_ = .001, *p* = .46, *η*_*p*_^*2*^ = .01; *F*_*2*_(1,94) = .42, *MS*_*E*_ = .002, *p* = .52, *η*_*p*_^*2*^ = .004.

### Discussion

To our surprise, we did not find a valence-to-spatial metaphoric congruency effect, whether or not participants performed the task simultaneously with a verbal WML. This might suggest that the activation of a spatial-valence metaphoric association only occurs in the mapping from spatial information to valence information, but not the other way around. The absence of the valence-to-spatial metaphoric congruency effect was not consistent with the results in Meier and Robinson’s [6] Study 2. Given that the power that we had to detect the effect was .84, we ruled out the possibility that the absence of the valence-to-spatial metaphoric congruency effect could be due to insufficient power. Before concluding the unidirectional nature of the spatial-valence metaphoric association, we attempted to further examine the valence-to-spatial metaphoric congruency effect by making changes in Experiment 2’s paradigm. In Experiments 3–5 we made one change on procedure or stimuli in each experiment to verify whether the valence-to-spatial metaphoric congruency effect would still be absent. In Experiment 6, we excluded the WML manipulation and followed exactly the same procedure as in Meier and Robinson’s [6] Study 2 to test whether their valence-to-spatial metaphoric congruency effect could be directly replicated.

## Experiments 3–5

Compared with Experiment 1, in which participants judged the valence of the target word by pressing a key, Experiment 2 required them to judge the valence of the prime word by saying aloud “positive” or “negative”. One could question whether the difference in the response modality of valence judgments would explain the discrepancy for the findings of these two experiments. To test this possibility, in Experiment 3 we adopted Experiment 2’s paradigm, except that the vocal response to the valence of the prime word was now replaced by key-press response. To further test the valence-to-spatial metaphoric congruency effect, in Experiments 4 and 5 we adopted Experiment 2’s paradigm but using positive and negative prime pictures (rather than positive and negative prime words) and using a visuospatial WML, 4-dot-position rehearsal task (rather than verbal WML), respectively.

### Methods

#### Participants

We recruited 49 (33 females; mean age = 19.83 years, *SD =* 1.69, 45 right-handers), 53 (23 females; mean age = 20.35, *SD =* 1.74, 51 right-handers), and 50 (27 females; mean age = 20.29, *SD =* 1.52, 47 right-handers) students in Experiments 3–5, respectively.

#### Stimuli and procedure

In Experiments 3–5, we used Experiment 2’s method, with only one change in each experiment. In Experiment 3, the vocal response to the valence of the prime word was changed to key-press response. The participants were instructed to press the p or q key to judge the positive or negative valence of the prime word, with key assignment being counterbalanced between participants.

In Experiment 4, positive and negative prime words were replaced by positive and negative prime pictures. We recruited 20 CUHK participants, who did not participate in any of the current experiments, to perform a normed rating task for 120 positive and 120 negative affective pictures, which were selected from the Chinese Affective Picture System [46]. Due to the high correlation between valence and arousal across 240 pictures (*r* = -.66), we could not match the arousal ratings for the 48 positive and 48 negative pictures that we selected for the current study. However, because the difference in valence ratings [4.99, *SD* = .18 vs. 2.07, *SD* = .21, *t*(94) = 72.30, *p*<.001] was much larger than that in arousal ratings [2.89, *SD* = .28 vs. 3.16, *SD* = .25, *t*(94) = 4.97, *p*<.001], the metaphoric congruency effect, if any, that occurred in this experiment would be more likely attributed to valence differences, rather than to arousal differences, between the positive and negative pictures.

In Experiment 5, the 6-digit rehearsal task was replaced by a 4-dot-position rehearsal task. The number of dots in this task was determined by a pilot study to make sure the task demand would not be too easy or too difficult. At the beginning of each WML block, participants were instructed to remember the sequence of 4 successive dots’ positions that were randomly presented in a 14 cm × 14 cm area and keep rehearsing their order overtly while performing valence judgment and p/q discrimination tasks. The four dots, measuring 0.5 cm in diameter, were randomly presented and each dot appeared on the screen for 2 s. At the end of each WML block, participants received a sheet on which four dots were printed in the positions that they appeared and were required to label their presentation order using the numerals 1, 2, 3, and 4 on the sheet. This task was modified from those in previous studies (e.g., [47]).

### Results

In Experiment 3, 1 participant’s data (female) were removed due to her low accuracy in the WML task (<80%). In Experiment 4, 5 participants’ data (2 females) were removed due to their low accuracies in the WML task (<80%) or their extreme overall RTs (±3 *SDs* from the mean). In Experiment 5, 10 participants’ data (3 females) were removed due to their low accuracies in the WML task (<80%) or their extreme overall RTs (±3 *SDs* from the mean). Data from the remaining participants were included in the final analyses (i.e., 48, 48, and 40 participants in Experiments 3, 4, and 5, respectively). [With *N* of 48, 48, and 40, in Experiments 3–5 and Cohen’s *d* of .54, which was computed based on the *F* value (8.07, i.e., *t* of 2.84) and sample size (28) reported in the Meier and Robinson’s [6] Study 2, the powers based on *α* = .05 (one-tailed) to detect the effect in our Experiments 3, 4, 5 were .84, .84, and .77 respectively.] The mean accuracy for participants’ digit recall (or dot position recall) in the WML blocks was .99 (*SD* = .03), .97 (*SD* = .06), and .98 (*SD* = .05) in Experiments 3–5, respectively.

The trimming procedure in Experiments 3, 4, and 5 was exactly the same as in Experiment 2. This procedure eliminated 5.0% (3.2% incorrect trials in p/q discrimination task and 1.8% of trials with RTs of 3 *SD*s beyond the overall RT mean in the valence judgment task), 5.5% (3.4% incorrect trials in p/q discrimination task and 2.2% of trials with RTs of 3 *SD*s beyond the overall RT mean in the vocal valence judgment task), and 4.1% (3.3% incorrect trials in p/q discrimination task and 0.8% of trials with RTs of 3 *SD*s beyond the overall RT mean in the vocal valence judgment task) of all responses in the RT analyses for Experiments 3, 4, and 5, respectively.

**Fig 4:**
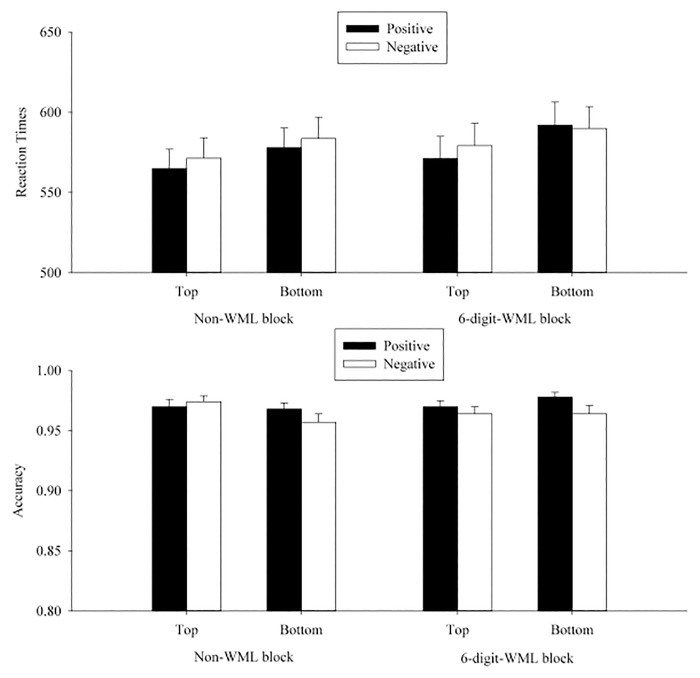
Mean RTs (upper panel) and accuracies (lower panel) in each condition on p/q discrimination in Experiment 3. The error bars indicated the standard errors of the means.

**Fig 5:**
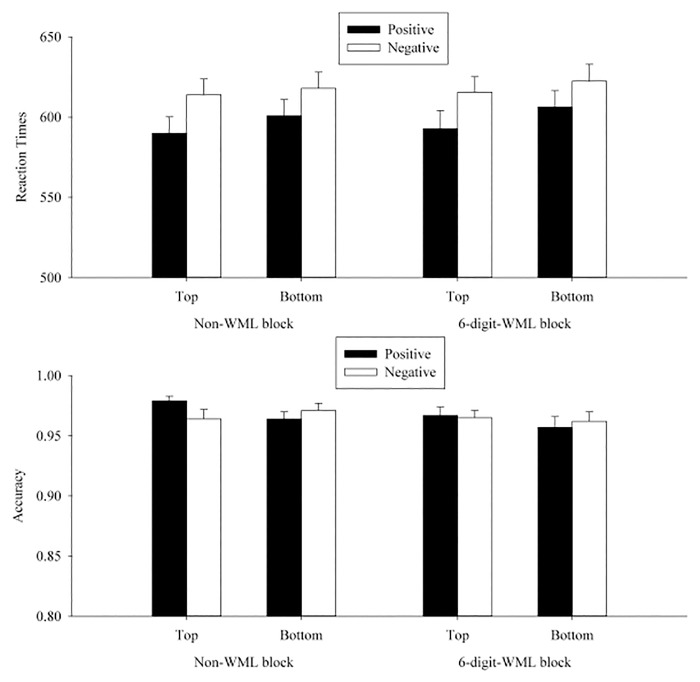
Mean RTs (upper panel) and accuracies (lower panel) in each condition on p/q discrimination in Experiment 4. The error bars indicated the standard errors of the means.

**Fig 6:**
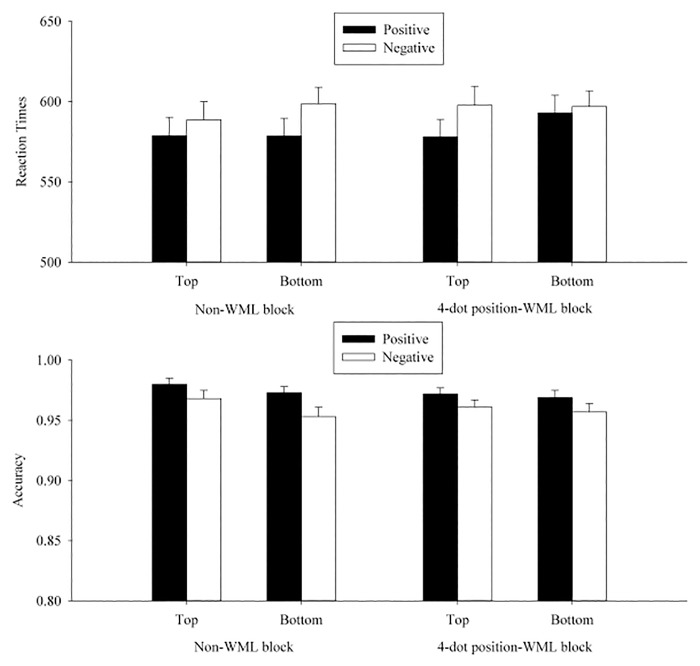
Mean RTs (upper panel) and accuracies (lower panel) in each condition on p/q discrimination in Experiment 5. The error bars indicated the standard errors of the means.

A 2 (WML: WML vs. non-WML) × 2 (position: top vs. bottom) × 2 (valence of the prime word: positive vs. negative) ANOVA was conducted for participants’ transformed RTs and accuracies separately for each experiment (see Tables 6–8 and Figs 4–6).

**Table 6:** Omnibus Analyses of Variance for log-transformed mean response times (RTs, in ms) and accuracies (in proportion) in Experiment 3.

	By participant								By item							
	RT				Accuracy				RT				Accuracy			
	*MS*_*E*_	*F*_*1*_	*p*	*η*_*p*_^*2*^	*MS*_*E*_	*F*_*1*_	*p*	*η*_*p*_^*2*^	*MS*_*E*_	*F*_*2*_	*p*	*η*_*p*_^*2*^	*MS*_*E*_	*F*_*2*_	*p*	*η*_*p*_^*2*^
**WML**	.001	7.21	.01	.13	.001	.22	.64	.005	<.001	11.98	.001	.11	.002	.13	.72	.001
**Position**	<.001	26.93	<.001	.36	.001	.43	.52	.009	<.001	33.03	<.001	.26	.002	.36	.55	.004
**Valence**	.002	.51	.48	.01	.001	4.30	.04	.084	<.001	1.14	.29	.01	.001	3.17	.08	.03
**WML × Position**	<.001	.51	.48	.01	.001	4.38	.04	.085	.001	.45	.50	.005	.001	3.03	.09	.03
**WML × Valence**	<.001	.17	.68	.004	.001	1.02	.32	.021	<.001	.04	.83	<.001	.002	.78	.38	.008
**Position × Valence**	<.001	1.42	.24	.03	.001	2.20	.14	.05	<.001	.93	.34	.01	.002	2.18	.14	.02
**WML × Position × Valence**	<.001	.82	.37	.02	.001	.16	.70	.003	.001	.44	.51	.005	.001	.16	.70	.002

*Note*: The degree of freedom in the participant analyses was (1, 47). The degree of freedom in the item analyses was (1, 94).

**Table 7:** Omnibus Analyses of Variance for log-transformed mean response times (RTs, in ms) and accuracies (in proportion) in Experiment 4.

	By participant								By item							
	RT				Accuracy				RT				Accuracy			
	*MS*_*E*_	*F*_*1*_	*p*	*η*_*p*_^*2*^	*MS*_*E*_	*F*_*1*_	*p*	*η*_*p*_^*2*^	*MS*_*E*_	*F*_*2*_	*p*	*η*_*p*_^*2*^	*MS*_*E*_	*F*_*2*_	*p*	*η*_*p*_^*2*^
**WML**	<.001	.85	.36	.02	.001	3.19	.08	.06	.001	.52	.47	.003	.001	1.80	.18	.01
**Position**	<.001	9.57	.003	.17	.002	1.22	.28	.03	.001	2.96	.09	.02	.001	1.01	.32	.01
**Valence**	<.001	52.04	<.001	.53	.002	.09	.77	.002	.001	12.14	.001	.06	.001	.06	.80	<.001
**WML × Position**	<.001	.41	.52	.01	.002	.10	.76	.002	.001	.05	.83	<.001	.001	.06	.80	<.001
**WML × Valence**	<.001	.05	.82	.001	.002	.35	.56	.01	.001	.01	.92	<.001	.001	.25	.62	.001
**Position × Valence**	<.001	3.78	.06	.07	.001	3.15	.08	.06	.001	.57	.45	.003	.001	1.80	.18	.01
**WML × Position × Valence**	<.001	.004	.95	<.001	.001	1.00	.32	.02	.001	<.001	.99	<.001	.001	.57	.45	.003

*Note*: The degree of freedom in the participant analyses was (1, 47). The degree of freedom in the item analyses was (1, 184).

**Table 8:** Omnibus Analyses of Variance for log-transformed mean response times (RTs, in ms) and accuracies (in proportion) in Experiment 5.

	By participant								By item							
	RT				Accuracy				RT				Accuracy			
	*MS*_*E*_	*F*_*1*_	*p*	*η*_*p*_^*2*^	*MS*_*E*_	*F*_*1*_	*p*	*η*_*p*_^*2*^	*MS*_*E*_	*F*_*2*_	*p*	*η*_*p*_^*2*^	*MS*_*E*_	*F*_*2*_	*p*	*η*_*p*_^*2*^
**WML**	.001	1.64	.21	.04	.001	.99	.33	.03	.001	2.44	.12	.03	.002	.78	.38	.01
**Position**	.001	3.21	.08	.08	.001	3.38	.07	.08	.001	5.08	.03	.05	.002	3.24	.08	.03
**Valence**	<.001	17.51	<.001	.31	.002	7.70	.01	.17	<.001	20.54	<.001	.18	.002	9.76	.002	.09
**WML × Position**	<.001	.09	.77	.002	.001	1.22	.28	.03	<.001	.02	.88	<.001	.001	.96	.33	.01
**WML × Valence**	<.001	.41	.53	.01	.001	.48	.49	.01	.001	.14	.71	.002	.002	.40	.53	.004
**Position × Valence**	<.001	.33	.57	.01	.001	.35	.56	.01	.001	.11	.74	.001	.002	.26	.61	.003
**WML × Position × Valence**	<.001	6.51	.02	.14	.001	.15	.71	.004	<.001	2.87	.09	.03	.001	.18	.68	.002

*Note*: The degree of freedom in the participant analyses was (1, 39). The degree of freedom in the item analyses was (1, 94).

In Experiment 3, the critical WML × position × valence interaction was not significant in RT, *F*_1_(1,47) = .82, *MS*_*E*_<.001, *p* = .37, *η*_*p*_^*2*^ = .02; *F*_2_(1,94) = .44, *MS*_*E*_ = .001, *p* = .51, *η*_*p*_^*2*^ = .005, or accuracy analyses, *F*_1_(1,47) = .16, *MS*_*E*_ = .001, *p* = .70, *η*_*p*_^*2*^ = .003; *F*_2_(1,94) = .16, *MS*_*E*_ = .001, *p* = .70, *η*_*p*_^*2*^ = .002. The position × valence interaction was also not significant in RT, *F*_1_(1,47) = 1.42, *MS*_*E*_<.001, *p* = .24, *η*_*p*_^*2*^ = .03; *F*_2_(1,94) = .93, *MS*_*E*_<.001, *p* = .34, *η*_*p*_^*2*^ = .01, or accuracy analyses, *F*_1_(1,47) = 2.20, *MS*_*E*_ = .001, *p* = .14, *η*_*p*_^*2*^ = .05; *F*_2_(1,94) = 2.18, *MS*_*E*_ = .002, *p* = .14, *η*_*p*_^*2*^ = .02.

Despite the high accuracy of participants’ digit recall in the WML blocks (99%), we conducted a 2 (position) × 2 (valence) ANOVA only on the data from the WML blocks in which digit recall was correct. In RT analyses, the main effect of position was significant, *F*_*1*_(1,47) = 26.47, *MS*_*E*_<.001, *p*<.001, *η*_*p*_^*2*^ = .36; *F*_*2*_(1,94) = 16.87, *MS*_*E*_<.001, *p*<.001, *η*_*p*_^*2*^ = .15. The main effect of valence was not significant, *F*_*1*_(1,47) = .40, *MS*_*E*_ = .001, *p* = .53, *η*_*p*_^*2*^ = .008; *F*_*2*_(1,94) = .58, *MS*_*E*_<.001, *p* = .45, *η*_*p*_^*2*^ = .006. The position × valence interaction was not significant, *F*_*1*_(1,47) = 2.30, *MS*_*E*_<.001, *p* = .14, *η*_*p*_^*2*^ = .047; *F*_*2*_(1,94) = 1.28, *MS*_*E*_<.001, *p* = .26, *η*_*p*_^*2*^ = .01. In accuracy analyses, the main effect of position was not significant, *F*_*1*_(1,47) = 1.20, *MS*_*E*_ = .001, *p* = .28, *η*_*p*_^*2*^ = .03; *F*_*2*_(1,94) = .91, *MS*_*E*_ = .001, *p* = .34, *η*_*p*_^*2*^ = .01. The main effect of valence was significant by participants, *F*_*1*_(1,47) = 5.12, *MS*_*E*_ = .001, *p* = .03, *η*_*p*_^*2*^ = .10; *F*_*2*_(1,94) = 3.36, *MS*_*E*_ = .002, *p* = .07, *η*_*p*_^*2*^ = .03. The position × valence interaction was not significant, *F*_*1*_(1,47) = .50, *MS*_*E*_ = .002, *p* = .48, *η*_*p*_^*2*^ = .01; *F*_*2*_(1,94) = .60, *MS*_*E*_ = .001, *p* = .44, *η*_*p*_^*2*^ = .006.

In Experiment 4, the critical WML × position × valence interaction was not significant in RT, *F*_1_(1,47) = .004, *MS*_*E*_<.001, *p* = .95, *η*_*p*_^*2*^<.001; *F*_2_(1,184)<.001, *MS*_*E*_ = .001, *p* = .99, *η*_*p*_^*2*^<.001, or accuracy analyses, *F*_1_(1,47) = 1.00, *MS*_*E*_ = .001, *p* = .32, *η*_*p*_^*2*^ = .02; *F*_2_(1,184) = .57, *MS*_*E*_ = .001, *p* = .45, *η*_*p*_^*2*^ = .003. The position × valence interaction was also not significant in RT, *F*_1_(1,47) = 3.78, *MS*_*E*_<.001, *p* = .06, *η*_*p*_^*2*^ = .07; *F*_2_(1,184) = .57, *MS*_*E*_ = .001, *p* = .45, *η*_*p*_^*2*^ = .003, or accuracy analyses, *F*_1_(1,47) = 3.15, *MS*_*E*_ = .001, *p* = .08, *η*_*p*_^*2*^ = .06; *F*_2_(1,184) = 1.80, *MS*_*E*_ = .001, *p* = .18, *η*_*p*_^*2*^ = .01.

Despite the high accuracy of participants’ digit recall in the WML blocks (97%), we conducted a 2 (position) × 2 (valence) ANOVA only on the data from the WML blocks in which the digit recall was correct. In RT analyses, the main effect of position was significant by participants, *F*_*1*_(1,47) = 8.36, *MS*_*E*_<.001, *p* = .006, *η*_*p*_^*2*^ = .15; *F*_*2*_(1,92) = 2.09, *MS*_*E*_ = .001, *p* = .15, *η*_*p*_^*2*^ = .02. The main effect of valence was significant, *F*_*1*_(1,47) = 28.30, *MS*_*E*_<.001, *p*<.001, *η*_*p*_^*2*^ = .38; *F*_*2*_(1,92) = 5.09, *MS*_*E*_ = .001, *p* = .03, *η*_*p*_^*2*^ = .05. The position × valence interaction was not significant, *F*_*1*_(1,47) = .36, *MS*_*E*_<.001, *p* = .55, *η*_*p*_^*2*^ = .008; *F*_*2*_(1,92) = .18, *MS*_*E*_ = .001, *p* = .67, *η*_*p*_^*2*^ = .002. In accuracy analyses, the main effect of position was not significant, *F*_*1*_(1,47) = .63, *MS*_*E*_ = .002, *p* = .43, *η*_*p*_^*2*^ = .01; *F*_*2*_(1,92) = .52, *MS*_*E*_ = .001, *p* = .47, *η*_*p*_^*2*^ = .006. The main effect of valence was not significant, *F*_*1*_(1,47) = .05, *MS*_*E*_ = .003, *p* = .83, *η*_*p*_^*2*^ = .001; *F*_*2*_(1,92) = .12, *MS*_*E*_ = .001, *p* = .73, *η*_*p*_^*2*^ = .001. The position × valence interaction was not significant, *F*_*1*_(1,47) = .34, *MS*_*E*_ = .003, *p* = .57, *η*_*p*_^*2*^ = .007; *F*_*2*_(1,92) = .28, *MS*_*E*_ = .001, *p* = .60, *η*_*p*_^*2*^ = .003.

In Experiment 5, RT analyses showed that the WML × position × valence interaction was significant by participants, *F*_1_(1,39) = 6.51, *MS*_*E*_<.001, *p* = .02, *η*_*p*_^*2*^ = .14; *F*_2_(1,94) = 2.87, *MS*_*E*_<.001, *p* = .09, *η*_*p*_^*2*^ = .03. Follow-up analyses showed that in the non-WML blocks, the valence × position interaction was not significant, *F*_1_(1,39) = 2.26, *MS*_*E*_<.001, *p* = .14, *η*_*p*_^*2*^ = .06; *F*_2_(1,94) = .67, *MS*_*E*_ = .001, *p* = .42, *η*_*p*_^*2*^ = .01, suggesting that there was no valence-to-spatial metaphoric congruency effect in the non-WML blocks. In the WML blocks, contrary to the findings of previous experiments, the valence × position interaction was significant by participants, *F*_1_(1,39) = 5.36, *MS*_*E*_<.001, *p* = .03, *η*_*p*_^*2*^ = .12; *F*_2_(1,94) = 2.23, *MS*_*E*_<.001, *p* = .14, *η*_*p*_^*2*^ = .02. Simple effect analyses showed that when the valence of the prime word was positive, participants’ responses in the p/q discrimination task were faster when the letter appeared at the top than at the bottom, *F*_1_(1,39) = 8.61, *p* = .006, *η*_*p*_^*2*^ = .18; *F*_2_(1,94) = 5.93, *p* = .02, *η*_*p*_^*2*^ = .06. When the valence of the prime word was negative, there was no difference whether the letter appeared at the top vs. bottom, *F*_1_(1,39) = .02, *p* = .90, *η*_*p*_^*2*^<.001; *F*_2_(1,94) = .10, *p* = .75, *η*_*p*_^*2*^ = .001. In accuracy analyses, neither the WML × position × valence interaction, *F*_1_(1,39) = .15, *MS*_*E*_ = .001, *p* = .71, *η*_*p*_^*2*^ = .004; *F*_2_(1,94) = .18, *MS*_*E*_ = .001, *p* = .68, *η*_*p*_^*2*^ = .002, nor the position × valence interaction was significant, *F*_1_(1,39) = .35, *MS*_*E*_ = .001, *p* = .56, *η*_*p*_^*2*^ = .01; *F*_2_(1,94) = .26, *MS*_*E*_ = .002, *p* = .61, *η*_*p*_^*2*^ = .003.

Despite the high accuracy of participants’ digit recall in the WML blocks (98%), we conducted a 2 (position) × 2 (valence) ANOVA only on the data from the WML blocks in which the dot position recall was correct. In RT analyses, the main effect of position was not significant, *F*_*1*_(1,39) = 2.89, *MS*_*E*_<.001, *p* = .10, *η*_*p*_^*2*^ = .07; *F*_*2*_(1,94) = 3.33, *MS*_*E*_<.001, *p* = .07, *η*_*p*_^*2*^ = .03. The main effect of valence was significant, *F*_*1*_(1,39) = 10.06, *MS*_*E*_<.001, *p* = .003, *η*_*p*_^*2*^ = .21; *F*_*2*_(1,94) = 6.40, *MS*_*E*_ = .001, *p* = .013, *η*_*p*_^*2*^ = .06. The position × valence interaction was significant by participants, (*F*_1_(1,39) = 5.21, *MS*_*E*_<.001, *p* = .03, *η*_*p*_^*2*^ = .12; *F*_2_(1,94) = 2.03, *MS*_*E*_<.001, *p* = .16, *η*_*p*_^*2*^ = .02). Simple effect analyses showed that when the valence of the prime word was positive, participants’ responses on p/q discrimination task were faster when the letter was presented at the top than at the bottom, *F*_1_(1,39) = 8.70, *p* = .005, *η*_*p*_^*2*^ = .18; *F*_2_(1,94) = 5.28, *p* = .02, *η*_*p*_^*2*^ = .053. When the valence of the prime word was negative, there was no significant difference whether the letter was presented at the top or at the bottom, *F*_1_(1,39) = .02, *p* = .89, *η*_*p*_^*2*^ = .001; *F*_2_(1,94) = .08, *p* = .78, *η*_*p*_^*2*^ = .001. In accuracy analyses, the main effect of position was not significant, *F*_*1*_(1,39) = .36, *MS*_*E*_ = .001, *p* = .55, *η*_*p*_^*2*^ = .009; *F*_*2*_(1,94) = .26, *MS*_*E*_ = .002, *p* = .61, *η*_*p*_^*2*^ = .003. The main effect of valence was not significant, *F*_*1*_(1,39) = 3.12, *MS*_*E*_ = .002, *p* = .09, *η*_*p*_^*2*^ = .07; *F*_*2*_(1,94) = 3.27, *MS*_*E*_ = .002, *p* = .07, *η*_*p*_^*2*^ = .03. The position × valence interaction was not significant, *F*_*1*_(1,39) = .24, *MS*_*E*_ = .001, *p* = .63, *η*_*p*_^*2*^ = .006; *F*_*2*_(1,94) = .14, *MS*_*E*_ = .002, *p* = .71, *η*_*p*_^*2*^ = .001.

### Discussion

Similar to Experiment 2, we did not find any valence-to-spatial metaphoric congruency effect in the non-WML or WML block whether response modality to the prime was key-press or vocal and whether the prime was a word or a picture. This suggests that the difference in response modality to valence judgments or in prime type (word vs. picture) in the primed valence judgment task cannot explain the absence of the valence-to-spatial metaphoric congruency effect. In Experiment 5, we still did not obtain any valence-to-spatial metaphoric congruency effect in non-WML blocks, but we unexpectedly found a significant valence-to-spatial metaphoric congruency effect when participants performed the main task and a dot-position recall task simultaneously. However, this finding should be interpreted with caution as it was inconsistent with the null effect in our previous four experiments.

Despite the sufficient statistical power to detect the effect, we still examine whether the absence of the valence-to-spatial metaphoric congruency effect was due to the small sample size in each of previous four experiments. We conducted a 2 (WML: WML vs. non-WML) × 2 (position: top vs. bottom) × 2 (valence of the prime word: positive vs. negative) ANOVA after collapsing the data across Experiments 2–5, with total *N* = 184. [Here we only report the results of AVOVA by taking participants as the random factor, because different stimuli were used in Experiments 2, 3, and 5 (words) and in Experiment 4 (pictures).] RT analyses showed that the main effects of block, position, and valence were all significant, *F*(1,183) = 4.30, *MS*_*E*_ = .001, *p* = .04, *η*_*p*_^*2*^ = .02; *F*(1,183) = 39.18, *MS*_*E*_<.001, *p*<.001, *η*_*p*_^*2*^ = .18; and *F*(1,183) = 52.17, *MS*_*E*_ = .001, *p*<.001, *η*_*p*_^*2*^ = .22. These suggested that participants responded faster in the non-WML blocks than in the WML blocks, when the letter appeared at the top of the screen than at the bottom, and faster when the letter was preceded by a positive word/picture than a negative word/picture. No interaction, including the critical valence × position × WML interaction, *F*(1,183) = 1.78, *MS*_*E*_<.001, *p* = .18, *η*_*p*_^*2*^ = .01, or the valence × position interaction, *F*(1,183) = 2.65, *MS*_*E*_<.001, *p* = .11, *η*_*p*_^*2*^ = .01, was significant. The position × valence interaction was also not significant in the non-WML blocks in this combined analysis, *F*(1,183) = .08, *MS*_*E*_<.001, *p* = .77, *η*_*p*_^*2*^<.001. Thus, even in the overall analyses with >180 participants, we did not obtain any evidence for the valence-to-spatial metaphoric congruency effect.

Still, given that the original experiment reported in Meier and Robinson’s [6] Study 2 did not have any WML manipulation, one could argue that the WML manipulation, despite its being done between blocks, might have modulated the valence-to-spatial metaphoric congruency effect. Hence, in our final experiment, we dropped the WML manipulation and attempted to directly replicate the findings reported in Meier and Robinson’s Study 2.

## Experiment 6

### Methods

#### Participants

Forty students (29 females; mean age = 20.20, *SD* = 1.49, 39 right-handers) were recruited. [With *N* of 40 in Experiment 6 and Cohen’s *d* of .54, which was computed based on the *F* value (8.07, i.e., *t* of 2.84) and sample size (28) reported in the Meier and Robinson’s [6] Study 2, the power based on *α* = .05 (one-tailed) to detect the effect in our Experiment 6 was .77.]

#### Stimuli and procedure

The stimuli and procedure were identical to Experiment 2, except that there was no WML manipulation—participants were not required to rehearse any 6-digit or 4-dot-position sequence in any blocks of trials.

### Results

**Fig 7:**
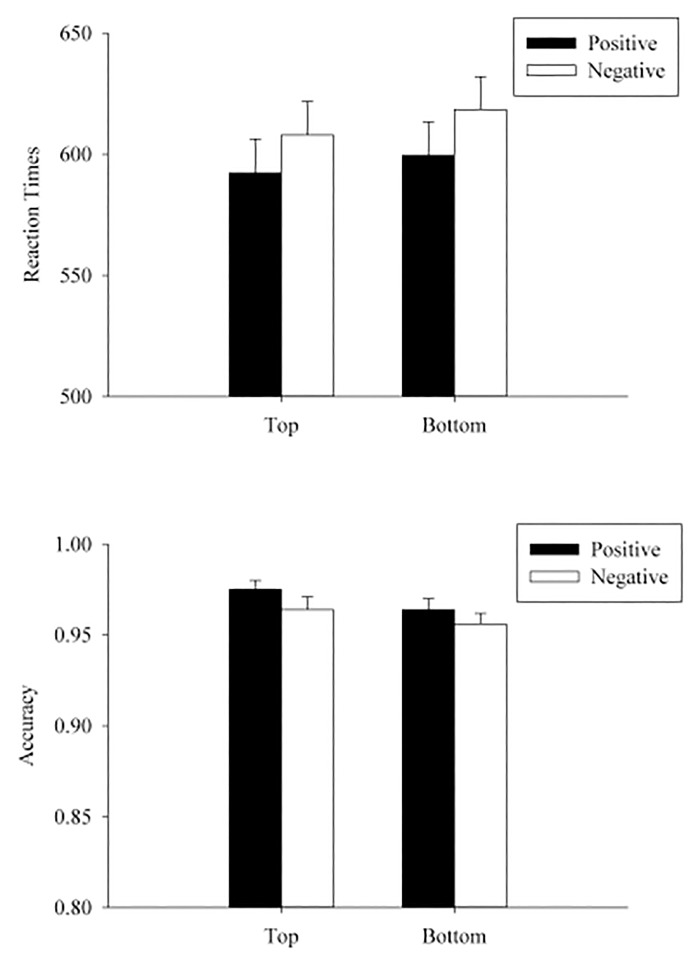
Mean RTs (upper panel) and accuracies (lower panel) in each condition on p/q discrimination in Experiment 6. The error bars indicated the standard errors of the means.

The trimming procedure was the same as in Experiment 2, which eliminated 5.2% of all responses (3.5% incorrect trials in p/q discrimination task and 1.7% of trials with RTs 3 *SD*s beyond the overall RT mean in the vocal valence judgment task). A 2 (position: top vs. bottom) × 2 (valence of the prime word: positive vs. negative) ANOVA was conducted for participants’ transformed RT and accuracy, separately (see Table 9 and Fig 7).

**Table 9:** Omnibus Analyses of Variance for log-transformed mean response times (RTs, in ms) and accuracies (in proportion) in Experiment 6.

	By participant								By item							
	RT				Accuracy				RT				Accuracy			
	*MS*_*E*_	*F*_*1*_	*p*	*η*_*p*_^*2*^	*MS*_*E*_	*F*_*1*_	*p*	*η*_*p*_^*2*^	*MS*_*E*_	*F*_*2*_	*p*	*η*_*p*_^*2*^	*MS*_*E*_	*F*_*2*_	*p*	*η*_*p*_^*2*^
**Position**	<.001	8.66	.01	.18	.001	6.81	.01	.15	.001	2.60	.11	.03	.001	4.92	.03	.05
**Valence**	<.001	25.48	<.001	.40	.001	4.62	.04	.11	.001	14.55	<.001	.13	.001	3.74	.06	.04
**Position × Valence**	<.001	.17	.68	.004	.001	.17	.68	.004	.001	.02	.88	<.001	.001	.14	.71	.001

*Note*: The degree of freedom in the participant analyses was (1, 39). The degree of freedom in the item analyses was (1, 94).

The critical position × valence interaction was not significant in RT or accuracy analyses [RT: *F*_1_(1,39) = .17, *MS*_*E*_<.001, *p* = .68, *η*_*p*_^*2*^ = .004; *F*_2_(1,94) = .02, *MS*_*E*_ = .001, *p* = .88, *η*_*p*_^*2*^<.001; accuracy: *F*_1_(1,39) = .17, *MS*_*E*_ = .001, *p* = .68, *η*_*p*_^*2*^ = .004; *F*_2_(1,94) = .14, *MS*_*E*_ = .001, *p* = .71, *η*_*p*_^*2*^ = .001], suggesting that there was no valence-to-spatial metaphoric congruency effect even when we used the same procedure of Meier and Robinson’s [6] Study 2.

### Discussion

In Experiment 6, we removed the WML manipulation in our paradigm, such that the design and procedure were highly similar to Meier and Robinson’s [6] Study 2. However, we still failed to obtain any valence-to-spatial metaphoric congruency effect. This suggests that the spatial-valence metaphoric association is not bidirectional. That is, while the processing of valence relies on spatial information, the reverse may not be true.

## General Discussion

The purpose of the current study was to test the directionality and automaticity of the activation of spatial-valence metaphoric association. We found the metaphoric congruency effect only in the spatial-to-valence direction, but not in the valence-to-spatial direction, consistent with the unidirectional view as proposed by the Conceptual Metaphor Theory (e.g., [1,2], see also [10,12] for a similar idea). Also, we found that the spatial-to-valence metaphoric congruency effect could be eliminated by verbal WML, suggesting that the spatial-to-valence metaphoric association may not be activated automatically.

### Unidirectionality of the spatial-valence metaphoric association

In Experiment 1’s non-WML blocks, spatial information (i.e., words were presented in the upper or lower position of the screen) could prime participants’ valence judgment of affective words; that is, their responses were faster when the spatial-to-valence relationship was congruent with the spatial-valence metaphoric association (positive/negative word at the top/bottom). This spatial-to-valence metaphoric congruency effect replicated the results of Meier and Robinson’s [6] Study 1. In Experiments 2–6, on each trial, participants made a valence judgment and then identified whether the letter that appeared at the top or bottom of the screen was p or q. Unlike the significant spatial-to-valence metaphoric congruency effect, the valence information (e.g., a positive word) did not activate the metaphorically congruent spatial information (e.g., upward), bias participants’ attention towards the top or bottom of the screen, and in turn trigger a valence-to-spatial metaphoric congruency effect in the p/q discrimination task. This was the case whether or not participants performed a concurrent verbal WML, whether response modality to the prime was key-press or vocal, and whether the prime was a word or a picture. This clearly suggests that the spatial-valence metaphoric association is unidirectional: whereas the spatial information has an effect on participants’ valence judgments, the valence information does not influence participants’ spatial attention and in turn, their responses in the p/q discrimination task. The absence of the valence-to-spatial metaphoric congruency effect was not consistent with the finding of Meier and Robinson’s [6] Study 2. It is noteworthy that the valence-to-spatial metaphoric congruency effect was not significant even after collapsing the data across multiple experiments, so we could rule out the possibility that the null effect was due to insufficient statistical power. Also, the absence of the valence-to-spatial metaphoric congruency effect was not due to the inclusion of the WML manipulation because the effect did not occur even when the exact same paradigm from Meier and Robinson’s Study 2 was used (Experiment 6).

It is noteworthy that the position was not fully counterbalanced with the WML variable in Experiments 1 and 4. That is, in Experiment 1 half of the positive/negative words was always presented at the top of the screen in the WML blocks and at the bottom of the screen in the non-WML blocks, whereas the other half, at the bottom of the screen in the WML blocks and at the top of the screen in the non-WML blocks. In Experiment 4, half of the positive/negative pictures was always followed by p/q appearing at the top of the screen in the WML blocks and at the bottom of the screen in the non-WML blocks, whereas the other half, followed by p/q appearing at the bottom of the screen in the WML blocks and at the top of the screen in the non-WML blocks. However, it should be emphasized that these two sets of stimuli did not significantly differ in various lexical variables: word length, *t*(94) = .24, *p* = .81, *d* = .04; Log HAL frequency, *t*(94) = .36, *p* = .72, *d* = .05; valence, *t*(94) = .17, *p* = .87, *d* = .02; arousal, *t*(94) = 1.21, *p* = .23, *d* = .18; familiarity, *t*(94) = .05, *p* = .96, *d* = .01; and concreteness, *t*(94) = 1.11, *p* = .27, *d* = .16. Therefore, we did not consider this would complicate the interpretation of the current findings.

One could argue whether the stimulus difference in the current study with those in Meier and Robinson [6] could account for this discrepancy. The stimuli we used were different from those in Meier and Robinson’s study since they were selected based on the normed ratings of participants drawn from the same pool as those in the present experiments. In addition, even though the task involved English stimuli in both studies, one could question that participants’ familiarity towards stimuli would have been different as Meier and Robinson’s participants performed the task in their first language, whereas our participants did so in their second language (i.e., English). As we have found a significant spatial-to-valence metaphoric congruency effect in Experiment 1’s non-WML blocks, as well as in Study 1b of Huang et al. [40], using the same set of stimuli and the same language requirement (i.e., in participants’ second language), it seems that the absence of valence-to-spatial metaphoric congruency effect might not be entirely attributed to stimulus difference or familiarity towards the stimulus language. Nevertheless, future researchers should directly compare the spatial-valence metaphoric congruency effects when the stimuli are presented in participants’ first language vs. their second language and verify whether valence words presented in different languages would lead to different types of processing of spatial-valence metaphoric association and whether this would be the case in both spatial-to-valence and valence-to-spatial directions.

The current study was designed to test the directionality, rather than the symmetry, of spatial-valence metaphoric association, so we did not intend to compare the relative strength of the metaphoric congruency effect in the two directions. Indeed, the paradigm difference in Experiments 1 and 2–6 could not allow a direct comparison between the spatial-to-valence and valence-to-spatial metaphoric congruency effects. While the two dimensions (space and valence) appeared as a compound cue in the target stimuli in Experiment 1, they were separately presented as prime and target in Experiments 2–6. As Meier and Robinson [6] discussed, Experiment 1 was a Stroop-like task, which presented the spatial and valence information simultaneously when participants made the judgments (positive/negative words were presented at the top or bottom), whereas Experiments 2–6 were priming tasks (i.e., whether the spatial attention was primed after the preceding valence judgment). Even though Meier and Robinson found that the valence judgment could prime the spatial attention in their Study 2, it was not the case in the present Experiments 2–6. Overall, our findings (significant spatial-to-valence metaphoric congruency effect and nonsignificant valence-to-spatial metaphoric congruency effect) could only support the unidirectional nature of spatial-valence metaphoric association.

The unidirectional findings of spatial-valence metaphoric association can also be accommodated by Santiago et al.’s [24] language usage account. (Note that they used the terms, bidirectionality/unidirectionality and symmetric/asymmetric, interchangeably, contrary to the distinction that we highlighted in the Introduction.) The authors considered various cases of metaphoric congruency effects, such as unidirectional spatial-time conceptual metaphors and bidirectional size-magnitude conceptual metaphors. They argued that the directionality of the metaphoric associations partially depends on language usage frequency (see [48,49] for a similar idea). If language usage shows an asymmetric pattern (e.g., people talk about time in terms of space much more often than space in terms of time), metaphoric congruency effects would be unidirectional. In contrast, if a symmetric pattern occurs in language (e.g., talking about number in terms of size as often as talking about size in terms of number), the metaphoric congruency effect would be bidirectional. According to this account, the spatial-valence metaphoric association is likely unidirectional because we could use spatial information to talk about affective states (e.g., “I am feeling low”), but we almost never use affective valence to talk about the vertical space (e.g., “I am going happier” to express the meaning of “up”).

Before concluding the unidirectionality of the spatial-valence metaphoric association, it is important to point out three potential concerns related to its interpretation. First, because of different tasks being used, participants were generally slower in Experiment 1’s valence judgment task than in Experiments 2–6’s p/q discrimination task. One could argue that the null valence-to-spatial metaphoric congruency effect in Experiments 2–6 was due to the genuine effect being masked by participants’ fast RT. To verify this, we divided participants in each of Experiments 2–6 into two groups based on the median split of their overall RT and examined whether a metaphoric congruency effect occurred in those with slower than median RT. To boost the statistical power in these analyses, we collapsed the data of the non-WML blocks in Experiments 2–5 and Experiment 6, and then conducted 2 (position: top vs. bottom) × 2 (valence: positive vs. negative) ANOVAs for participants with faster and slower than median RTs, respectively. (Even though we split the participants into two sets based on their RTs, the ANOVAs were also conducted on their log-transformed RTs.) The results did not show any position × valence interaction for participants with faster than median RT [*F*(1,110) = 1.12, *MS*_*E*_<.001, *p* = .29, *η*_*p*_^*2*^ = .01] or those with slower than median RT [*F*(1,111) = .06, *MS*_*E*_<.001, *p* = .81]. Thus, the absence of valence-to-spatial metaphoric congruency effects was unlikely attributable to faster RTs in the p/q discrimination task, relative to those in the valence judgment task in Experiment 1. To test this further, we performed analyses on participants’ mean RT in the slowest bin of their RT distributions. Specifically, we divided the individual participant’s RT with each condition into six bins by following the procedure of previous studies (e.g., [50]). Individual RT data within each condition is first sorted from fastest to slowest in terms of responses. The first 16.7% of the data is then averaged, followed by the second 16.7%, and so on. This method allows a more direct examination of the RT data, relative to other analytic procedures, without assuming any particular shape of the RT distribution, such as ex-Gaussian (e.g., [51]). Then, we conducted a 2 (position) × 2 (valence) repeated-measures ANOVA on this subset of data after collapsing the data of non-WML blocks in Experiments 2–5 and 6. Results showed an absence of the position × valence interaction, *F*(1,223) = .16, *MS*_*E*_ = .06, *p* = .69, *η*_*p*_^*2*^ = .001, so there was no valence-to-spatial metaphoric congruency effect even in the slowest bin of participants’ RT in the current study. Overall, we conclude that the valence-to-spatial metaphoric congruency effect did not occur in the current study and might not depend on participants’ RTs.

Second, whereas valence is the “target” information being judged in Experiment 1, vertical position (spatial information) is not the “target” information being judged in Experiments 2–6, where participants needed to discriminate a letter (p or q) that appeared in the metaphorically congruent or incongruent position. One could argue that Experiments 2–6 only tested how the valence information could bias participants to attend to a specific spatial direction, which in turn influenced their response in the p/q discrimination task. However, even though the p/q discrimination task was not a direct measurement of spatial information, it was certain that this task could test the valence-to-spatial metaphoric congruency effect. If valence information does (or does not) have an influence on the processing of spatial information, the preceding valence judgment would (or would not) prime the activation of metaphorically congruent vertical position (up or down), and in turn, the p/q discrimination task would (or would not) be faster when the letter appeared in that position. While the difference in the two paradigms might make it difficult to compare the magnitudes of the spatial-to-valence and valence-to-spatial metaphoric congruency effects, it is noteworthy that, as mentioned above, we did not intend to compare the magnitudes of the two effects across our experiments (i.e., to test the symmetry vs. asymmetry of spatial-valence metaphoric association). Rather, we focused on whether the metaphoric congruency effects occurred in the experiments that were designed to examine each of them (i.e., to test the unidirectionality vs. bidirectionality of spatial-valence metaphoric association).

On the other hand, one could argue that the absence of valence-to-spatial metaphoric congruency effect might be attributed to the paradigm difference in Experiments 1 and 2–6. In Experiment 1, the spatial information was concurrently presented with valence information (i.e., as a compound cue that was often used in interference paradigms, such as the Stroop task). In contrast, in Experiments 2–6, the valence information was a prime, followed by a target (i.e., the spatial p/q discrimination task). Thus, the absence of valence-to-spatial metaphoric congruency effect might be limited to this type of priming paradigm. It should be noted that studies, which showed the bidirectionality effects, did not always use the conceptually identical tasks to examine the effects in both directions (such as upward-powerful [16] and cleanliness-morality [17]). Nevertheless, it is important to test whether both spatial-to-valence and valence-to-spatial metaphoric congruency effects would occur in future studies when their magnitudes would be directly compared in conceptually identical tasks that are designed to test the directionality/symmetry of spatial-valence metaphoric association.

Third, one could argue whether the metaphoric congruency effect was attributed to semantic associations, rather than to metaphoric associations. Metaphoric associations refer to the cross-domain mappings from concrete concepts to abstract concepts. People could not directly interact with abstract concepts because they do not have physical referents in the real world. Rather, they need to represent abstract concepts in terms of sensorimotor information originating from concrete concepts. Semantic association refers to concepts that are related semantically or associatively. For example, doctor-nurse is associatively related because people most likely respond *nurse* when they are asked to freely associate a word related to *doctor*. To examine whether the current pattern of results could be attributed solely to semantic associations, we used two methods to quantify the semantic associations between spatial concepts and valence concepts.

The first way to quantify the spatial-valence semantic association is via Nelson et al.’s [52] free association norm, a database that provides values of associative strength between two words. This norm was developed by having participants associate words (i.e., targets) freely in response to specific cue word. The higher the value of the associative strength between the cue and the target, the higher the proportion of participants who responded that target in response to the cue. The free association norm can be downloaded at http://link.springer.com/content/esm/art:10.3758/BF03195588/file/MediaObjects/Nelson-BRM-2004.zip. We first paired up each of the affective words stated in S1 Materials with a spatial word associated with upward or downward meaning (above/below, high/low, over/under, top/bottom, or up/down). Then, we checked the forward (cue-to-target) and backward (target-to-cue) associative strengths in Nelson et al.’s norm. (The spatial word was regarded as the cue and the affective word was regarded as the target.) Based on the listing of Nelson et al.’s norm, only 6 out of 96 affective words (angel, depression, dirt, failure, heaven, hell) had an associative strength with one spatial word. In other words, most of the affective words were not associated to any spatial words, so we assigned zero associative strength for these other pairs. The mean forward and backward associative strengths across all affective words were very low [.0005 (*SD* = .0025) and .0004 (*SD* = .0025), respectively]. Thus, the metaphoric congruency effect we obtained in the current study was unlikely attributed to the associative strength between the spatial concepts and the valence concepts.

The second way to quantify the spatial-valence semantic association is via semantic similarity, as defined by Latent Semantic Analysis (LSA) [53]. As the LSA cosine reflects the degree of association in natural language between words—that is, how well one word may fit into the same passage as the other and the extent to which one word may substitute for the other in text—it can be used to quantify the interitem association between the spatial and valence concepts. Following the lead of previous studies (e.g., [54,55]), we obtained the LSA cosine for all of the spatial word-affective word pairs through the pairwise comparison function via http://lsa.colorado.edu/. We then computed the mean LSA cosine for each affective word. A 2 (spatial concept: up vs. down, within-item variable) × 2 (valence word: positive vs. negative, between-item variable) mixed-factor ANOVA was conducted on these mean LSA cosines. Results showed that the main effect of spatial concept was significant, *F*(1,94) = 218.38, *MS*_*E*_<.001, *p*<.001, *η*_*p*_^*2*^ = .70, but the main effect of valence word was not, *F*(1,94) = 2.09, *MS*_*E*_ = .007, *p* = .15. The interaction was significant, *F*(1,94) = 14.46, *MS*_*E*_<.001, *p*<.001, *η*_*p*_^*2*^ = .13. Simple effect analyses showed that the difference between the LSA cosine of *UP*-positive pairs (mean = .19, *SD* = .06) and *DOWN*-positive pairs (mean = .14, *SD* = .05) was significant, *F*(1,94) = 172.60, *MS*_*E*_<.001, *p*<.001, *η*_*p*_^*2*^ = .65, and the difference between the LSA cosine of *UP*-negative pairs (mean = .16, *SD* = .07) and *DOWN*-negative pairs (mean = .13, *SD* = .05) was also significant, *F*(1,94) = 60.23, *MS*_*E*_<.001., *p*<.001, *η*_*p*_^*2*^ = .39. If semantic association played a critical role in the spatial-to-valence metaphoric congruency effect, we would obtain a similar ordinal pattern of LSA cosine and metaphoric congruency effects. Following the pattern of LSA cosine, one would predict a positive metaphoric congruency effect for positive words—positive words were responded to faster when they appeared at the top of the screen than at the bottom and a *reversed* metaphoric congruency effect for negative words—negative words were also responded to *faster* when they appeared at the top than at the bottom. These were not quite consistent with our findings. In Experiment 1’s non-WML blocks, only the former, but not the latter, was true, so the semantic association may not fully explain the spatial-to-valence metaphoric congruency effect. Moreover, semantic association, as defined by LSA cosines, is bidirectional, which may not accommodate the current findings that the spatial-valence metaphoric association is unidirectional. Overall, whether the semantic associations were quantified by Nelson et al.’s [52] associative strengths or by Landauer et al.’s [53] LSA cosines, the current findings could not be solely attributed to the semantic associations between spatial and valence concepts.

### Automaticity of the activation of spatial-valence metaphoric association

In Experiment 1 we found the spatial-to-valence metaphoric congruency effect in the non-WML blocks, but the effect was eliminated in the WML blocks. This suggests that the activation of the metaphoric association is attention demanding, thereby not fully in line with the criteria of automaticity [31,56]. This result was in contrast to the view that the concrete domain is activated automatically when thinking about abstract concepts.

Previous studies (e.g., [6]) considered the metaphoric congruency effect to be automatic because it was a Stroop-like effect. Given that the levels of information in the concrete and abstract domains were fully crossed in the experiments, the concrete domain (e.g., vertical position) could not provide participants any information about the decision being made in the abstract domain (e.g., word valence), so that participants would have no incentive to process the information in the concrete domain. Nevertheless, the responses to the abstract domain were still influenced by prior task-irrelevant information from the concrete domain. This was similar to the Stroop interference effect that the color-word meaning (which is irrelevant to the task demand) is still activated even when the task only requires responses to the ink color of the color word (see [57] for a review). Hence, Meier et al. [21] argued that under this circumstance, the occurrence of metaphoric congruency effects could suggest that concrete concepts are activated obligatorily, providing evidence for the automaticity of the activation of the metaphoric association.

However, using the dual-task technique to distinguish automatic from non-automatic processes, the current experiments suggested that Meier and Robinson’s [6] conclusion might not be always correct. The assumption underlying this technique is that only non-automatic processes, but not automatic processes, could be influenced by the demand of a secondary task (i.e., WML, see [29,33,34,37]). In the current study, we showed that the spatial-to-valence metaphoric congruency effect could be eliminated by verbal WML, suggesting that the activation of the spatial-to-valence metaphoric association is attention demanding (i.e., a non-automatic process). Despite being consistent with the conclusion of some previous studies [25,26], this might not be consistent with the Conceptual Metaphor Theory [1,2,20].

Given that a verbal WML task could eliminate the spatial-to-valence metaphoric congruency effect in Experiment 1, one could argue whether the activation of the spatial-to-valence metaphoric association might depend on verbal processing (see [58,59] for studies that used similar verbal WML tasks to test whether language plays a role in perceptual color processing). However, because we did not test whether the spatial-to-valence metaphoric congruency effect could be eliminated by nonverbal WML, it is not clear whether nonverbal processing might also be involved in the activation of spatial-to-valence metaphoric association. This has to be tested in future studies by adapting the non-verbal WML task in our Experiment 1. Nonetheless, for the purpose of the current study, we clearly showed that the metaphoric congruency effect could be disrupted by verbal WML, which was sufficient to conclude that the metaphoric association between space and valence may not be automatically activated.

### Implications of the current findings for the polarity account [60,61]

While the current experiments were not designed to test the alternative accounts of metaphoric congruency effects, it is important to discuss whether our findings might be better accommodated by one of them, the polarity account [60], which was based on Proctor and Cho’s [61] general account of polarity correspondence. According to Lakoff and Johnson’s [2] Conceptual Metaphor Theory, the spatial-to-valence metaphoric congruency effect refers to the finding that positive words are judged faster when they appear at the top than at the bottom *and* that negative words are judged faster when they appear at the bottom than at the top. Thus, the valence × position interaction occurs with a symmetric RT pattern for positive and negative words. However, the current findings were only consistent with the prediction for positive words, but not for negative words (see Fig 2). This pattern was reported using similar paradigms in previous studies (e.g., [60,62,63]) and was said to be accommodated better by the polarity account [60,61]. According to this account, dimensions consist of polar opposites, such as good-bad and up-down. Each dimension has a default endpoint (+polar) and the corresponding opposite endpoint (-polar) [61]. The +polar endpoint of a dimension has a processing advantage over the-polar endpoint [64,65]. Lakens [60] summed four processing benefits relating to the spatial-valence metaphoric congruency effect. Two of them are related to the two dimensions of stimuli. Specifically, people process the positive word faster than the negative word, and words presented at the top position are processed faster than those presented at the bottom position. The third one is related to the response codes. In a bimanual response task, responses are usually coded as YES/NO, TRUE/FALSE, or POSITIVE/NEGATIVE. Responses are faster when they are coded as +polar (yes, true, or positive) than-polar (no, false, or negative) [66]. The fourth one is the polarity correspondence principle [61], which states that responses are faster when the polarities of the stimulus’ conceptual and perceptual dimensions overlap (positive words presented at the top and negative words at the bottom) than when they do not overlap. After summing together these four kinds of the processing benefits, the RT pattern is expected as follows: positive stimuli are responded to faster when they are presented at the top than at the bottom of the screen; but for negative stimuli, they are expected to be responded to equally fast no matter where they are presented—top or bottom.

This asymmetric RT pattern predicted by the polarity account is different from the RT pattern predicted by the Conceptual Metaphor Theory; that is, the valence × position interaction should show a symmetric RT pattern for positive and negative words. The spatial-to-valence metaphoric congruency effect in Experiment 1’s non-WML blocks (i.e., positive words were responded to faster when appearing at the top than at the bottom, whereas there was no RT difference for negative words when they appeared at the top vs. bottom) was more congruent with the polarity account than with the Conceptual Metaphor Theory. It is noteworthy that the polarity account might not explain all of the findings related to the metaphoric association in the literature, some of which did show a symmetric pattern of response bias in a task that did not use RT as the dependent measure. For instance, Lundholm [67] asked participants to draw a line to express their affective status and showed that the lines for positive status were in a relatively upper position, whereas those for negative status, in a relatively lower position. Wapner et al. [68] found that, after receiving positive (or negative) feedback (e.g., A or F grade) for their performance, participants showed an upward (or downward) shift when bisecting a luminous square. Crawford et al. [69] reported that positive (or negative) stimuli were better remembered when they were encoded in the upper (or lower) position on a screen. All these findings were better accommodated by Conceptual Metaphor Theory than by the polarity account, suggesting that it is premature to reject the former theory, while in favor of the latter account.

Nevertheless, unlike the Conceptual Metaphor Theory, which could accommodate the unidirectionality, but not non-automaticity, of the activation of spatial-valence metaphoric association, the polarity account might not accommodate the unidirectionality or non-automaticity of the spatial-valence metaphoric association demonstrated in the current study. First, in Proctor and Cho’s [61] comprehensive review of the evidence that supported the polarity account, such as orthogonal stimulus-response compatibility (e.g., Simon effect), they argued that the RT benefit is always present whenever the polarities in the conceptual and perceptual dimensions overlap, suggesting that the polarity benefits could automatically occur (see also [70,71]). This is not in line with the current findings that the activation of the spatial-to-valence metaphoric association was not automatic. Second, the polarity account might not predict the unidirectional activation of the metaphoric association either. According to Lakens [60], “…the polarity correspondence principle does not predict the asymmetrical influence of the perceptual (source) dimension on the conceptual (target) dimension, which is a theoretical assumption in conceptual metaphor theory (Lakoff & Johnson, 1999)” (page 735). In other words, the polarity account could predict the bidirectional effects in both the spatial-to-valence and valence-to-spatial metaphoric congruency effects, with the following patterns: for the spatial-to-valence direction, the congruency effect occurs for positive words, but not for negative words; for the valence-to-spatial direction, the congruency effect occurs when the letter appears at the top, but not at the bottom of the screen.

Nevertheless, it is important to note that the polarity account was initially developed to explain the findings that involved compound cue stimuli (e.g., both spatial and valence information presented as a single stimulus, as in our Experiment 1), but not those that involved prime and target stimuli (e.g., spatial and valence information separately presented, as in our Experiments 2–6). As a result, this account might not necessarily predict the presence (or absence) of the valence-to-spatial congruency effect in our Experiments 2–6. Future studies should test this account by using compound cue stimuli to trigger a potential valence-to-spatial congruency effect.

### Implications of the current findings for the Coherent Working Models Theory

According to Santiago et al.’s [12] Coherent Working Models Theory, the directionality of metaphoric congruency effects depends on the level of activation of the conceptual dimensions, which could be modulated by many factors, such as attentional orientation. Consistent with this view, Santiago et al. [24] reported that spatial information could influence participants’ valence judgments when their attention is directed to the spatial dimension, whereas the valence information could affect participants’ spatial judgments when their attention is directed to the valence dimension. Regarding the present findings, in Experiment 1 we presented three successive fixations to orient participants’ attention to the location of the forthcoming positive/negative words. Hence, the findings that the primed spatial information had an influence on participants’ subsequent valence judgments (i.e., the spatial-to-valence metaphoric congruency effect) were consistent with the Coherent Working Models Theory. However, in Experiments 2–6, even though participants needed to judge the valence information (i.e., their attention being directed to the valence dimension) before doing the p/q discrimination task, the valence information did not affect the spatial attention to the top or bottom of the screen, and in turn failed to modulate the responses in the p/q discrimination task. Thus, the unidirectionality of the spatial-valence metaphoric association seems not to be consistent with the Coherent Working Models Theory. One possibility is that the activation level of valence information was not high enough to impact the spatial attention and in turn the p/q discrimination, yet this could not explain why the same set of stimuli did yield the congruency effect in the other direction (i.e., the spatial-to-valence metaphoric congruency effect). On the other hand, the non-automaticity of the activation of spatial-to-valence metaphoric association could be accommodated by the Coherent Working Models Theory. Santiago et al. [24] showed that the metaphoric congruency effect did not occur when attention was not directed to the source dimension. Similarly, when participants’ attention was divided to perform the verbal WML task, they did not show any spatial-to-valence metaphoric congruency effect in Experiment 1, thereby showing the necessity of attentional resource in producing the metaphoric congruency effect. In short, Santiago et al.’s Coherent Working Models Theory could partially account for the present findings.

## Conclusion

The present study addressed two issues related to the activation of the spatial-valence metaphoric association, directionality and automaticity. Based on the findings of six experiments, we conclude that the activation of spatial-valence metaphoric association is unidirectional; that is, it occurs in the spatial-to-valence direction, but not in the valence-to-spatial direction. Whether the absence of the valence-to-spatial congruency effect might be due to participants’ overall RT difference in the current study and in Meier and Robinson’s [6] Study 2 awaits further investigation. The activation of spatial-to-valence metaphoric association does not occur automatically because the metaphoric congruency effect could be impaired by the WML. While the unidirectional nature of the spatial-valence metaphoric association was consistent with the Conceptual Metaphor Theory, the non-automatic nature of this association might contradict what the Conceptual Metaphor Theory would predict. Besides, it is noteworthy that a number of metaphoric associations were reported to be bidirectional, such as size-magnitude (e.g., [24]) and spatial-powerfulness (e.g., [16]). Future studies should further investigate the factors that determine the directionality of a metaphoric association, such as language usage [24] as well as examine the two directions of the spatial-valence metaphoric association by using two conceptually identical tasks. It is also important to test whether the alternative accounts of Conceptual Metaphor Theory, such as the polarity account [60] and the Coherent Working Models Theory [12], could be modified to account for the non-automaticity and unidirectionality of the spatial-valence metaphoric association.

## Supporting Information

S1 Materials The 48 positive and 48 negative words used in the present study and the 10 spatial words used in the additional analyses of the semantic associations between the spatial words and affective words. (DOCX)

S1 Data Raw data (including erroneous responses and outlier RT responses) of all experiments. (XLSX)
