# Supplementary material for: Re-Examining the Automaticity and Directionality of the Activation of the Spatial-Valence "Good is Up" Metaphoric Association
Source: PLoS One. 2015 Apr 13;10(4):e0123371. doi: 10.1371/journal.pone.0123371 (PMC4395106; doi:10.1371/journal.pone.0123371)
Supplement: S1 Materials — (DOCX) [file pone.0123371.s001.docx]

**Materials S1**

**The 48 positive and 48 negative words used in the present study and the 10 spatial words used in the additional analyses of the semantic association between the spatial words and affective words.**

| Positive words | Comfort, victory, optimism, excellence, kiss, hug, enjoyment, warmth, heaven, adventure, honey, sweetheart, bless, birthday, pride, passion, liberty, loyalty, beach, improvement, perfection, confidence, wedding, champion, reward, wisdom, honesty, delight, health, desire, respect, kindness, wealth, talent, holiday, vacation, excitement, hope, glory, humor, welfare, miracle, acceptance, angel, paradise, joy, achievement, success | |
| --- | --- | --- |
| Negative words | Danger, penalty, disappointment, suicide, devil, selfishness, accident, disaster, fault, trouble, murderer, anger, cancer, loneliness, greed, thief, evil, dirt, funeral, punishment, injury, loss, sickness, nightmare, destruction, theft, tragedy, guilt, killer, mistake, ignorance, illness, poverty, depression, failure, crisis, loser, bankrupt, stress, death, hunger, hell, fear, starving, discomfort, pain, pollution, headache | |
| Spatial concepts with upward meaning | | up, top, above, over, high |
| Spatial concepts with downward meaning | | down, bottom, below, under, low |
